# Accounting for aid: estimating the impact of United States’ global health investments on mortality among women of reproductive age using synthetic control and Bayesian methods

**DOI:** 10.7189/jogh.15.04067

**Published:** 2025-03-21

**Authors:** Karar Zunaid Ahsan, Gustavo Angeles, Allysha Choudhury, Kavita Singh, Tory M Taylor, Farhan Majid, Rachel Lucas, Robert L Cohen, Atul A Gawande, William Weiss

**Affiliations:** 1 Department of Public Health Leadership and Practice, Gillings School of Global Public Health, University of North Carolina at Chapel Hill, Chapel Hill, North Carolina, USA; 2 Data for Impact (D4I), Carolina Population Center, University of North Carolina at Chapel Hill, Chapel Hill, North Carolina, USA; 3 Department of Maternal and Child Health, Gillings School of Global Public Health, University of North Carolina at Chapel Hill, Chapel Hill, North Carolina, USA; 4 Public Health Institute, Oakland, California, USA; 5 Office of the Assistant Administrator, Bureau for Global Health, U.S. Agency for International Development, Washington, DC, USA; 6 Ariadne Labs at Brigham & Women's Hospital and Harvard TH Chan School of Public Health, Boston, Massachusetts, USA; 7 Department of International Health, Johns Hopkins University, Baltimore, Maryland, USA

## Abstract

**Background:**

The United States government (USG) is a key global actor in preventing mortality and supporting lifesaving health services among women and children in low- and middle-income countries (LMICs). Since the Foreign Assistance Act of 1961, USG has funded global health programmes targeting specific conditions and strengthening health systems for the delivery of essential services via the United States Agency for International Development (USAID) and other USG agencies. However, directly quantifying and attributing the impact of USG health investments is challenging due to the inability of conducting randomised control trials for such large programmes at scale. In this study, we compared two quasi-experimental impact evaluation approaches to estimate the effects of sustained USG health investments on mortality among women of reproductive age (WRA).

**Methods:**

We employed synthetic control analysis and a Bayesian alternative to synthetic control to estimate the impact of USG’s global health investments on WRA mortality rate across 16 LMICs that received sustained, multifaceted, and above-average USAID global health funding levels from 2005 to 2019.

**Results:**

Countries receiving sustained, multifaceted, and above-average USAID global health funding had a reduction in the annual WRA mortality rate by 0.65 deaths per 1000 WRA throughout the post-treatment period. For the years (*i.e.* 2009–2019) where the effect estimates are statistically significant (*P* < 0.001), the reduction in WRA mortality rate was 0.80 deaths per 1000 WRA. Sensitivity analyses and Bayesian modelling supported the robustness of these findings. We conservatively estimated that about 1.0–1.3 million WRA deaths were averted in study countries between 2009 and 2019 as a result of USG health investments.

**Conclusions:**

Our results suggest that well-funded donor initiatives can substantially reduce WRA mortality rates beyond what would have been achieved without such investments. Sustained donor investments significantly reduce WRA mortality, underscoring the transformative potential of well-funded global health initiatives. Our study also demonstrates that synthetic control and Bayesian models are valuable tools for evaluating the impact of large-scale global health financing.

The United States government’s (USG) commitment to improving the overall health and well-being of women and children has been instrumental in improving coverage and quality of essential health services in low- and middle-income countries (LMICs) [1]. Through deploying robust funding from the American people, the USG – including the United States Agency for International Development (USAID) – supports strengthening health systems, including an increasing emphasis on primary health care, as key to delivering equitable, high-quality care [1]. Emphasising integration within these systems promises enhanced resilience and long-term health improvements for women and children worldwide [2]. Recognising the imperative of this cause, the combined efforts and learned experiences of the last decade have been pivotal in improving the lives of women and children globally [3].

In low-income countries, HIV-related conditions are the leading cause of death among women of reproductive age (WRA, ages 15–49), followed by maternal causes, cancer, cardiovascular disease, other non-communicable diseases, injuries, tuberculosis (TB), malaria, pneumonia, and other infectious diseases [4]. The leading causes were similar in the year 2000, except for cancer and cardiovascular diseases becoming more common as progress has been made against communicable diseases [5]. Though the incidence of cancer is currently lower in LMICs compared to high-income countries, the number of deaths from cancer is higher in LMICs [6,7]. The United Nations Sustainable Development Goals (SDGs) are a call to action for all countries. Among these goals, SDG 3 aims to ‘ensure healthy lives and promote well-being for all at all ages.’ This goal includes specific targets for maternal mortality, under-five mortality, and neonatal mortality, as well as for other leading causes of mortality, including HIV and AIDS, TB, malaria and neglected tropical diseases, non-communicable diseases, injuries, and substance abuse [8].

Regarding maternal mortality, Banchani and Swiss (2019) found that targeted reproductive health aid had a slight but significant impact on maternal mortality for 130 LMICs between 1996 and 2015, after controlling for gross domestic product (GDP) per capita, skilled birth attendant coverage, adolescent fertility rate, and contraceptive prevalence (modern methods) [9]. Pickborn and Ndikumana (2016) found that health aid contributed to reductions in maternal mortality from 1975 to 2010 in 75 LMICs [10]. The effect of health aid was significant both with and without controls for the initial Human Development Index (HDI) and initial GDP per capita. Other studies have demonstrated mortality impacts from the President’s Emergency Relief Plan (PEPFAR) and the Global Fund to Fight AIDS, Tuberculosis, and Malaria on total mortality and adult mortality [11,12]; however, no study has yet examined the impact of health aid on all-cause mortality among WRA.

The USG is the largest donor globally of development assistance for health [13,14]. In 2017 alone, for example, the American people provided USAID over five billion USD to support health programmes in 96 countries to address a broad range of global health areas, including HIV and AIDS, maternal and child health, family planning and reproductive health, malaria, nutrition, global health security, and TB. These health areas are important contributors to the mortality of children under five and women ages 15–49 in these countries. Assessing whether USG funding contributes to declines in mortality for these populations is imperative for evaluating its programmes. A recent study found that the under-5 mortality rate declined from 1999–2016 approximately twice as fast in countries with sustained USG funding compared to an otherwise comparable set of countries [15]. Here, we examine how USG’s health assistance impacted the mortality of women of reproductive age.

## METHODS

### Theory of change

To quantify the impact of USG’s global health investment on WRA mortality, we hypothesised that USG funding for a broad range of health areas and partnership with host governments, development partners, and other stakeholders would benefit WRA and thus accelerate mortality reductions among women in countries with consistent, broad, and large USG investment. Therefore, the mortality among WRA in those countries would be quantifiably lower than if the USG had not invested in those countries over the last two decades.

Among USG agencies, USAID provides health assistance to LMICs across the broadest range of health areas as compared to other USG agencies: HIV/AIDS, family planning, maternal and child health, nutrition, vulnerable children, TB, malaria, neglected tropical diseases, and pandemics [16]. Given the breadth of this programming, this paper primarily relies on USAID approaches to describe the overall USG theory of change for global health programming. USAID specifically has three strategic global health priorities: preventing child and maternal deaths, combating infectious diseases, and controlling the HIV and AIDS epidemic [17]. While the first strategic priority explicitly targets reducing mortality among WRA who are pregnant, women in LMICs also benefit from health services and health systems strengthening initiatives under the remaining strategic priorities [17]. Countries that receive sustained and high levels of funding across multiple health programme areas, in theory, are expected to have a lower WRA mortality rate than would otherwise have occurred without USAID (and other USG partner agencies) engagement and support. As these three strategic priorities have been operationalised, USAID health programmes for maternal and child health, nutrition, family planning, tuberculosis, malaria, neglected tropical diseases, HIV and AIDS, and other programmes would decrease WRA mortality in LMICs through the following common approaches:

1) Providing access to quality health care services: USAID aims to eliminate or control specific infectious diseases by sustainably strengthening the health care system to deliver multiple specific disease-targeting interventions. Expanding quality medical and reproductive health services should lower death rates among women and children, ensuring they receive essential care to maintain health and combat diseases. USAID-supported efforts focus on training more health care professionals, especially in areas with inadequate services, and bolstering the overall quality and accessibility of medical care.

2) Promoting healthy behaviours: health initiatives promote women’s well-being, decreasing mortality rates through education on healthy living and encouraging the use of preventive measures like vaccinations and health screenings. USAID supports these efforts at the country level.

3) Strengthening the health care system: to effectively address WRA mortality, a comprehensive strategy is essential, focusing on improving health system infrastructure, health workforce density, and securing essential commodities via a robust supply chain. USAID plays a pivotal role by championing more investment from various development partners, aiding in buying vital medical supplies, advancing quality care methods, and encouraging policies for improved health sector leadership and governance.

4) Evaluating and adapting programmes: continuous monitoring and improvement of health programmes is vital to ensure that women receive the health services they need to reduce preventable mortality. Investing in tools like Demographic and Health Surveys, impact evaluations, and robust health information systems is essential for developing data-driven strategies and adaptive management of health programmes to accelerate the reduction of mortality rates among WRA.

5) Influencing national policy and leveraging resources and interest from others: USAID funds and actively works to shape health policies in its partner countries, moving beyond a passive donor role to create cross-sector partnerships. Its method of catalysing contributions from public, private, and NGO sectors enriches health programmes with diverse expertise and investments. This broad engagement enhances programme efficacy and encourages more donor and stakeholder participation, leading to synergies in local health programmes.

**Figure 1:**
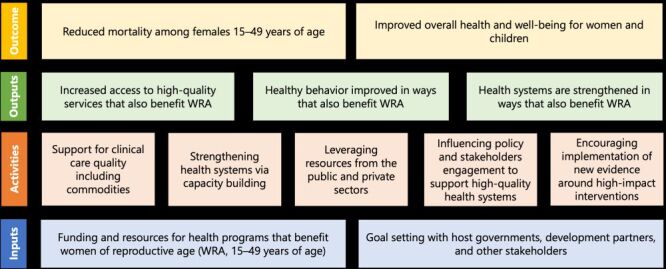
Theory of change for the United States government’s health investment in low- and middle-income countries.

The hypothesised theory of change framework for reducing mortality among WRA is depicted in **Figure 1**.

### Synthetic control analysis (SCA)

The SCA is a comparative case study method that provides a quantitative estimate of the impact of an intervention, also called a ‘treatment effect’, by comparing a treated unit to a synthetic counterfactual [18]. This approach is appropriate in the following situations: there are no readily available control units; there are a small number of treatment units; the intervention is not randomly assigned; and the treatment effect can take time to present itself. The methodological approach of SCA involves constructing a ‘synthetic control’ from a weighted combination of comparison countries selected from countries that have not been exposed to the programme being evaluated (*i.e.* donor pool) since no country from the donor pool can be directly used as the appropriate counterfactual. This synthesis is achieved by defining a pre-treatment and a treatment period and then by aligning outcome and explanatory variables from the pre-treatment phase of these potential comparison countries with those of the countries receiving the treatment. In this process, certain countries may be excluded due to significant pre-treatment differences, while others contribute variably to the synthetic control [19,20]. For effective SCA application, it is crucial that the synthetic control’s pre-treatment outcome trajectory closely aligns with those of the treatment country, enabling a comparative analysis of outcomes in the treatment period to assess the impact of the treatment. Divergence in outcomes between the synthetic control and the treatment unit during the treatment period indicates the treatment’s effect [18,21]. The SCA uses a placebo test to infer the significance of the treatment effects [18,22].

### Bayesian dynamic multilevel latent factor model

While the SCA can be a suitable method for producing an unbiased, comparable counterfactual for a treatment unit, extensions of the synthetic control method have proliferated in recent literature [23]. These alternative methods build on the logic of SCA to predict outcomes for counterfactuals of treatment units but can have improved predictive performance, increased model robustness, and relaxed assumptions to produce a causal estimate and yield an interpretable uncertainty interval. One of these methods is a Bayesian dynamic multilevel latent factor model (BM), which adopts a Bayesian causal inference framework to provide a counterfactual to treatment units and estimate treatment effects with uncertainty intervals [24]. It can address multiple sources of heterogeneity and dynamics present in the data, incorporates the use of latent factor terms, and allows covariate coefficients to vary by unit or over time. The BM method requires a large number of pre-treatment time points (*i.e.* years) and a greater number of donor units than treatment units to estimate the treatment effect accurately. More information on the Bayesian model is included in Text S1 in the **Online Supplementary Document**.

### Treatment year

We analysed funding data from USAID for 101 LMICs between 1999 and 2019. It should be noted that since income-based country classifications are reviewed annually and can change, we have used the country classifications from the year 2000 for selecting treatment and donor countries in this analysis [25]. Our goal was to identify when USAID’s global health investments substantially increased. After reviewing USAID’s global health financing trends (Figure S1 in the **Online Supplementary Document**), we found a sharp rise in funding after 2004 (no publicly available funding data are available for 2005–2006 due to various factors, including the Iraq War and the initiation of major initiatives such as PEPFAR in 2003). As a result, we selected 2005 as the treatment year for our SCA. Apart from reviewing the overall health investments by USAID, we also analysed country-level financing for health programmes during this period and ascertained that most countries witnessed a significant rise in aggregate funding post-2004 (figures not shown).

### Treatment unit

We purposely selected the countries to comprise the treatment unit. We selected countries for the treatment unit based on two key criteria:

1) the country received funding for a broad range of health areas, including reproductive, maternal, and child health, as well as infectious diseases (*e.g.* not just for HIV or HIV/TB);

2) the country received a relatively large amount of sustained funding.

As in other analyses [15], since we do not have a known threshold for funding that would result in a significant impact in USG-funded countries as compared to those same countries without USG funding, we test our theory of change here at the margin: if we do not find an impact among the countries that we believe best align with our theory of change, we would not expect to find an impact in other countries. For practical purposes, we selected the countries for the treatment unit using information from USAID financial systems as USAID provides health assistance to LMICs across the broadest range of health areas as compared to other USG agencies: HIV/AIDS, family planning, maternal and child health, nutrition, vulnerable children, TB, malaria, neglected tropical diseases, and pandemics [16].

While reviewing the country-level data on USAID financing, we found that 45 countries received substantial funding annually. As the treatment unit, we identified countries that received USAID funding every year between 2007 and 2019, for a broad range of health areas (*e.g.* not just for HIV or HIV/TB). We then selected a subset with both above-median total (*i.e.* 45 557 USD per year) and above-median per-capita (*i.e.* 2.73 USD per year) funding for health programmes from USAID (both figures are in the current US dollar). These 16 countries (Figure S2 in the **Online Supplementary Document**) constituted our treatment unit for SCA: Afghanistan, Cote d’Ivoire, Ethiopia, Haiti, Kenya, Malawi, Mali, Mozambique, Nigeria, Rwanda, Senegal, South Africa, Tanzania, Uganda, Zambia, and Zimbabwe. Our treatment unit was created by combining data from all 16 countries, effectively weighting it according to each country’s population.

### Countries to constitute ‘donor pool’

The SCA requires that units from the donor pool do not receive exposure to the treatment. Thus, all countries classified by the World Bank as low- or middle-income countries at the beginning of USAID funding data (*i.e.* 1999 / 2000) that did not receive above-median total and per-capita funding for health programmes from USAID (Figure S2 in the **Online Supplementary Document**), and had a population of 500 000 or larger before the treatment year (*i.e.* 2004), were considered eligible to be donors. Our study included countries in the donor pool that received some USAID funding during the study period. We opted not to exclude countries with USAID funding during the treatment period to ensure a sufficiently large donor pool for a robust analysis. This approach, allowing countries with a relatively low level of USAID funding, was chosen for its conservative nature and alignment with our theory of change that significant and sustained funding is necessary to effect change. Our criteria initially allowed for the inclusion of 77 low- or middle-income countries in the donor pool.

The rationale for considering countries with a population greater than 500k for the donor pool was to ensure the data’s robustness and reliability, which are critical for accurate statistical analysis. Larger populations provide more stable and generalisable data, which is essential when creating a synthetic control intended to simulate the counterfactual scenario for a treatment unit. Smaller countries, which are often small islands, might not provide a robust enough data set (*e.g.* data not available for key variables) for accurate analysis and could introduce volatility that undermines the validity of the synthetic control. Therefore, including countries with larger populations helps minimise these issues and ensures that the data-driven approach of the SCA can be applied effectively. Our criteria to this point allowed 59 low- or middle-income countries to be included in the donor pool. Among these 59 countries, 23 countries showed an ascending trend in the percentage of WRA deaths among total deaths during the pre-treatment period and remained in our donor pool (Figure S3 in the **Online Supplementary Document**). The criteria of ‘ascending trend in proportion of WRA deaths’ was considered because the treatment unit showed an ascending trend in WRA mortality during the pre-treatment period (Figure S4 in the **Online Supplementary Document**). From this pool, Montenegro was dropped because it was established in 2006, and Turkmenistan, Iraq, and Turkey were dropped because no HIV data were available as another criterion for admission to the donor pool. This left 19 countries that were used for the donor units and 16 countries as treatment units in the analyses (Figure S5 in the **Online Supplementary Document**). The selection process for the donor pool is described in Text S2 in the **Online Supplementary Document**.

### Outcome variable

For SCA, we focused on the following outcome variable:



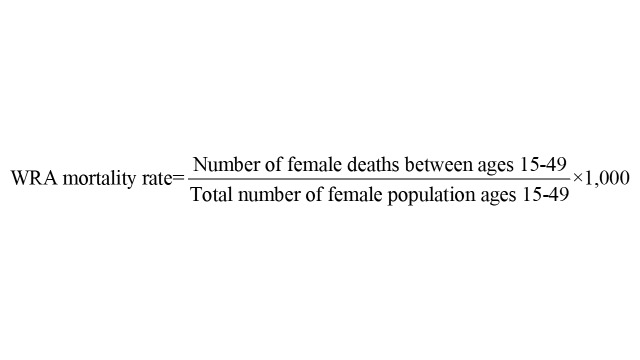



Reducing mortality among women of reproductive age in developing countries is an expected outcome across most USG global health programmes and is critical for numerous reasons. First, it emphasises well-being beyond maternal mortality by assessing mortality caused by reproductive and non-reproductive issues. While nearly 95% of all maternal fatalities occur in LMICs [5], women also die younger in LMICs from both chronic and infectious diseases, reflecting inadequate health care system efficacy and accessibility [26–28]. Second, women’s well-being is strongly linked to that of their families and communities. Their premature deaths can destabilise family structures, worsen poverty, and adversely affect child health and education [29]. Third and finally, investing in women’s health bolsters economic productivity. Improving maternal health enables women to participate in the labour force more effectively and propels economic growth [30]. Thus, addressing high mortality in this population is imperative not only for human rights and equity but also for strategically fostering sustainable societal development.

The trajectory of the outcome variable shows that the WRA mortality rate for the treatment unit continued to increase during 1990–2001 [31], plateaued at 7.9 deaths per 1000 WRA for a few years, and then decreased to 4.3 deaths per 1000 WRA in 2019, in line with our theory of change. For many donor countries, except Sudan, Eritrea, Congo (Brazzaville), Mongolia, etc., a gradual increase (or plateauing) in the WRA mortality rate can also be observed during 1990–2001 (Figure S4 in the **Online Supplementary Document**).

### Predictors for SCA

Many factors affect women’s health and their rights and societal roles, including economic, environmental, social, political, and demographic characteristics. A detailed literature review, which helped to identify a range of predictors of WRA, was organised into several mutually reinforcing areas to improve the health, dignity, and well-being of women and children on the basis of the Global Strategy for Women’s, Children’s, and Adolescents’ Health 2016–2030 [32]. We selected 12 indicators considering these areas and the respective rationale for inclusion in the model (**Table 1**). The definitions of the indicators are provided in Table S1 in the **Online Supplementary Document**.

**Table 1:** Final set of predictors for synthetic control analysis

Serial No.	Indicators	Policy area	Data source
1.	HIV prevalence, total (% of population ages 15–49)	Service delivery	WDI
2.	Tuberculosis case detection rate	Service delivery	WDI/GTC 2010
3.	Contraceptive prevalence rate (any method)	Service delivery	WDI/Alkema et al. (2013)
4.	Immunisation coverage, measles	Service delivery	WDI
5.	Net non-USG ODA received per capita (current USD)	Financing	OECD
6.	Mean years of schooling for females	Human development	UNDP
7.	% urban population	Population	WDI
8.	Total fertility rate	Population	WDI
9.	Total population (logged)	Population	WDI
10.	Female labour force participation	Human development	WDI
11.	GDP per capita	Financing	WDI
12.	Polity5 score*	Political stability	INCSR

GDP – gross domestic product, HIV – human immunodeficiency virus, ODA – overseas development assistance, USG – The United States government

*Polity5 – policy score by INSCR (Table S1 of the **Online Supplementary Document**).

### Sensitivity analyses

In addition to comparing the findings of a traditional SCA with those of a BM, we also carried out several sensitivity analyses to test the calculations and inferences of our main analyses. For sensitivity analyses, we carried out traditional SCAs by dropping South Africa (scenario 2) and Nigeria (scenario 3) from the treated unit. The rationale for excluding South Africa from the treatment unit as a sensitivity analysis was that its cause of death and burden of diseases were highly skewed toward HIV and AIDS, and it received substantial HIV-related funding from USG sources (viz., PEPFAR) compared to non-USG donors, unlike the other countries in our treatment unit. The rationale for excluding Nigeria from the treatment unit as a sensitivity analysis was based on several factors: Nigeria has the largest population among the countries in the treatment unit, giving it the potential to influence the aggregated mortality estimates of the treatment unit; and it falls on the borderline of median per capita funding (Figure S2 in the **Online Supplementary Document**) for selection into the treatment unit. Furthermore, we conducted a residual regression analysis following methodologies proposed by Arkhangelsky et al. (2021) and Clarke et al. (2023) to examine the role of non-USG funding in our findings as a part of our sensitivity analyses [33,34].

## RESULTS

### The optimised SCA model

**Figure 2:**
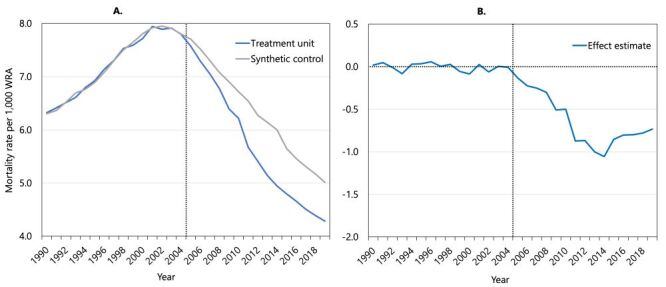
Trends in mortality rate among women of reproductive age (**Panel A**) from the optimised synthetic control analysis and effect estimates (**Panel B**) during the study period.

The SCA’s empirical optimisation procedure selected the best weighting of countries from the donor pool to create a synthetic control, minimising the difference between WRA mortality trends in the treated unit and the synthetic control during the pre-2005 period. Our SCA model’s fit was excellent (RMSPE = 0.0451168) (**Figure 2**, Panel A). The resulting synthetic control was a weighted average of the outcomes in the Central African Republic (37%), Chad (26%), Equatorial Guinea (11%), Gambia (11%), Niger (6%), Gabon (4%), Sudan (3%), and Guinea-Bissau (2%). The selection of countries to construct the synthetic control in this study was a mixture of low-income (Central African Republic, Chad, Gambia, Niger, Sudan, and Guinea-Bissau) and upper-middle-income (Equatorial Guinea, Gabon) countries from the sub-Saharan Africa region but sharing similar characteristics of having increasing WRA mortality during the pre-2000 period, with a peak around 2003. Overall, the comparison of predictors used for SCA indicated a good resemblance of pre-intervention predictors between the treatment unit and its synthetic control (Table S2 in the **Online Supplementary Document**). During the post-intervention period, the mortality rate among WRA in the treatment group of countries decreased noticeably faster than the synthetic control (**Figure 2**, Panel A). The gap in WRA mortality rate between the countries receiving above-average global health funding and its synthetic control is shown by the effect estimates in **Figure 2**, Panel B.

To assess the statistical significance of the disparity between the treatment unit and the constructed synthetic control, we conducted a placebo test to determine if the observed difference might simply be a product of random variation [18]. This involves comparing the treatment effects estimated by SCA with what might occur if each country in the donor pool were randomly assigned as the treatment unit instead of the actual countries that received substantial health investments from USG (our current treatment unit). The placebo test yields a range of differences in WRA mortality rates by comparing the synthetic control with every country in the donor pool not included in the treatment unit. If we observe that these other countries’ mortality differences relative to the synthetic control are similar, we conclude that there is not enough statistical evidence to assert that the treatment unit (*i.e.* countries with both broad and high USG health investment) has a notably lower mortality rate among WRA compared to the control. The results of this placebo testing are shown in Figure S6 in the **Online Supplementary Document**. From this placebo test, we see that the effect estimates became statistically significant (*P* < 0.001) after 2008 (**Table 2**). The average treatment effect is a reduction in the annual mortality rate by 0.65 deaths per 1000 WRA throughout the post-treatment period. For the years where the effect estimates are statistically significant (2009–2019), the reduction in mortality rate is 0.80 deaths per 1000 WRA.

**Table 2:** Effect estimates from the optimised synthetic control analysis model and their *P*-values

Year	Effect estimate	*P*-value	Standardised *P*-value*
2005	−0.1302	0.579	0.105
2006	−0.2259	0.526	<0.001
2007	−0.2526	0.526	0.053
2008	−0.3026	0.474	0.105
2009	−0.5077	0.368	<0.001
2010	−0.4981	0.421	<0.001
2011	−0.8717	0.263	<0.001
2012	−0.8661	0.263	<0.001
2013	−1.0007	0.263	<0.001
2014	−1.0565	0.211	<0.001
2015	−0.8516	0.316	<0.001
2016	−0.8020	0.316	<0.001
2017	−0.7989	0.316	<0.001
2018	−0.7794	0.316	<0.001
2019	−0.7321	0.368	<0.001
Post-treatment average	−0.6451	0.368	<0.001

*P-value adjusted after accounting for pre-treatment matching quality.

### Bayesian model

**Figure 3:**
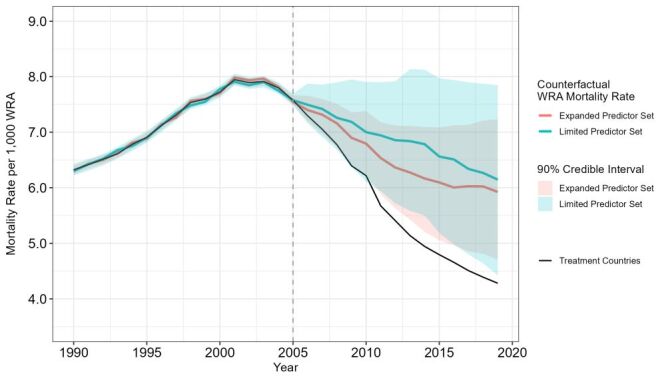
Bayesian model inferences of estimated effect with 90% credible intervals

Using the same covariates and set of treatment and control countries, the BM found similar treatment effects on changes in WRA mortality rate compared to the SCA. The model posterior distribution showed an average post-treatment effect of −1.29 deaths (90% credible interval (CI) = −0.18, −2.40), which translates to an estimated decrease in the mortality rate of 1.3 deaths per 1000 WRA in the selected countries. The estimated treatment effects were statistically significant after 2010 and throughout the remainder of the treatment period, as indicated by credible intervals excluding zero, suggesting a progressively increasing influence of global health investments on WRA mortality rates over time (**Figure 3**).

We also ran the BM with an expanded predictor set that included additional variables of ART usage, female HIV prevalence, physician density, adolescent fertility rate, average life expectancy, human development index, net national income per capita, and maternal mortality ratio. The results of the BM with the expanded predictor set resulted in a more conservative but still significant effect size (−0.96 deaths; 90% CI = −0.18, −1.73), with all treatment effects remaining significant after 2008, translating to an estimated 0.96 deaths decrease in WRA mortality rate per 1000 WRA in the selected countries (**Table 3**).

**Table 3:** Treatment effects over time using the Bayesian model

Year	Limited predictor model			Expanded predictor model		
	**Treatment Effect**	**90% Credible Interval**		**Treatment Effect**	**90% Credible Interval**	
		**Lower Bound**	**Upper Bound**		**Lower Bound**	**Upper Bound**
2005	−0.01	0.10	−0.12	−0.01	0.08	−0.09
2006	−0.22	0.13	−0.58	−0.13	0.10	−0.36
2007	−0.34	0.14	−0.80	−0.23	0.08	−0.55
2008	−0.52	0.09	−1.12	−0.34	0.04	−0.73
2009	−0.78	0.02	−1.55	−0.49	−0.03	−0.96
2010	−0.81	0.11	−1.69	−0.58	−0.01	−1.16
2011	−1.26	−0.27	−2.23	−0.86	−0.21	−1.51
2012	−1.42	−0.31	−2.51	−0.97	−0.22	−1.71
2013	−1.73	−0.45	−3.00	−1.13	−0.29	−1.97
2014	−1.87	−0.56	−3.18	−1.20	−0.26	−2.15
2015	−1.78	−0.39	−3.17	−1.27	−0.26	−2.29
2016	−1.81	−0.33	−3.28	−1.38	−0.31	−2.46
2017	−1.84	−0.29	−3.39	−1.48	−0.34	−2.62
2018	−1.85	−0.25	−3.48	−1.62	−0.42	−2.82
2019	−1.82	−0.14	−3.57	−1.69	−0.42	−2.95
Post-treatment average	−1.29	−0.18	−2.40	−0.96	−0.18	−1.73

There was substantial variation in treatment effects by country (Figure S7 in the **Online Supplementary Document**). Countries that showed a substantial and significant treatment effect include Mozambique, South Africa, Tanzania, Uganda, and Zambia. However, some countries that faced natural disasters or conflict after 2005 had a higher mortality rate compared to a counterfactual, for example, Haiti and Afghanistan. Other countries like Kenya, Malawi, Mali, and Nigeria also had small or negative treatment effects or wide credible intervals that overlapped the counterfactual WRA mortality rate with the observed WRA mortality rate.

### Sensitivity analyses

For sensitivity analyses, we carried out SCA by dropping South Africa (scenario 2) and Nigeria (scenario 3) from the treatment unit. The estimated effect sizes are slightly larger in scenario 2 and noticeably larger in scenario 3 and clearly statistically significant (the standardised *P*-value for post-treatment years was <0.001) as compared to the original scenario with both South Africa and Nigeria included (Figure S8 in the **Online Supplementary Document**). For the detailed results of the sensitivity analyses, please see Text S3 in the **Online Supplementary Document**.

Similarly, in the BM, when Nigeria was dropped from the data set, there was a greater but still significant effect size for the limited predictor set (−1.73 deaths; 90% CI = −0.72, −2.71) and for the expanded predictor set (−1.31 deaths; 90% CI = −0.50, −2.13). Similarly, when South Africa was dropped from the data set, the BM showed a decreased but significant effect size for the limited predictor set (−1.19 deaths; 90% CI = −0.20, −2.18) and for the expanded predictor set (−0.56 deaths; 90% CI = 0.83, −2.00).

For the BM, robustness checks were also run to assess if model assumptions were violated. A placebo test where the pre-treatment period is divided into two halves and the model is trained on the first half to predict the second half can suggest the robustness of the BM estimator if the second half is predicted and actual trends are aligned. Results from a placebo test should result in a low and non-significant treatment effect, as there is no actual treatment taking place during this period. Thus, the results of the placebo test suggested that the expanded predictor set BM (−0.48 deaths; 90% CI = −1.58, 0.63) has a better model fit and robust estimator than the limited predictor set BM (0.84 deaths; 90% CI = 0.13, 1.54).

We also examined the role of non-USG funding in our findings as a part of our sensitivity analyses. Our theory of change suggests that USG funding may stimulate additional non-USG funding and complement it. However, non-USG funding could also confound our results. Despite similarities between our treatment and comparison groups during the pre-treatment period, time-varying changes – such as global efforts to meet the United Nations Millennium Development Goals around the time USAID and its USG partners increased its funding (circa 2005) – may have influenced both USG and non-USG funding.

We address non-USG funding in two ways. First, in our baseline analysis, we control for non-USG funding per capita within the SCA framework, which selects and weights treatment and comparison groups to ensure pre-treatment similarity. As shown in **Figure 2**, our main results remain robust even after adjusting for non-USG funding. Second, we conducted a residual regression analysis following methodologies proposed by Arkhangelsky et al. (2021) and Clarke et al. (2023) [33,34], which helps control for confounding effects of non-USG funding during the treatment period. This analysis further isolates the impact of USG funding from non-USG funding. The analysis is based on a two-step procedure. Step 1 involves estimating a residual WRA mortality indicator that is net of any effects from non-USG funding per capita (an Ordinary Least Squares regression of WRA mortality on USG funding per capita is run to estimate residual WRA mortality). Step 2 takes the residual WRA mortality indicator, uses this residual as the outcome variable, re-runs the exact same SCA and Bayesian analysis as reported for baseline models, and reports a ‘residual average treatment effect (ATE).’ Text S4 in the **Online Supplementary Document** explains this procedure in more detail.

Our main findings show that the residual ATE is slightly higher than baseline estimates in both Bayesian and SCA models, indicating that non-USG funding is not the primary driver of our results (Table S7 in the **Online Supplementary Document**). We calculated the ratio of the ATE from the residual model (which controls for non-USG funding) to the overall model (which includes both USG and non-USG effects) as an estimate of how much of the total effect is attributable to non-USG funding. A ratio of 0 suggests that the observed effects are primarily due to non-USG funding or USG funding working through its influence on non-USG funding, while a ratio of 1 indicates that non-USG funding does not bias our main results. Ratios between 0 and 1 suggest a partial influence of non-USG funding, whereas ratios greater than 1 imply that non-USG funding may be biasing the baseline ATE estimates downward.

The ratio of residual ATE to base ATE is greater than 1 for our main findings from both SCA (1.04) and Bayesian models (1.35), suggesting that our main findings may be partially biased downwards due to non-USG funding. This finding is robust, as removing Nigeria – an outlier due to significant non-USG funding – does not alter our results. The ratio remains at 1.04 for the SCA model. Although the ratio is lower than 1.35 for the Bayesian model, the ratio for the sample excluding Nigeria is still 1.02, which is slightly above 1 and very similar to the SCA model. However, excluding South Africa, a country with substantial HIV-related USG funding compared to non-USG donors [35,36], lowers the ratio in the SCA to 0.87 and to 0.72 in Bayesian analysis, suggesting non-USG funding also contributes to effects (13–28% of overall ATE) in other countries. When considering Bayesian analysis for countries excluding South Africa, the lower bound of the confidence interval for the residual regression overlaps with 0, indicating that when South Africa is excluded from the treatment group of countries, we cannot rule out a contribution of non-USG funding to the treatment effects observed, either directly or due to the catalytic influence of USG funding.

## DISCUSSION

Mortality among WRA is a critically important indicator of a country’s overall health status and health system effectiveness. In LMICs, governments, donors, international organisations, nongovernmental organisations, and community-based organisations have made large investments to improve vulnerable populations’ survival, health, and well-being. Thus, it is important to assess the effect of such funding on under-five mortality and mortality among WRA, and also to understand which types of investments lead to the greatest impacts. In a recent study [15], we provided evidence that adequately funded USG programs helped countries to reduce child mortality to significantly lower rates than would have occurred without those investments. In this paper, we focus on WRA, defined as ages 15 to 49 [37]. Our SCA findings showed that countries receiving above-average global health funding had significantly (*P* < 0.001) lower mortality rates among WRA than the synthetic control. We applied results from the SCA and expanded predictor BM to populations of WRA to estimate the number of WRA lives saved. We estimated that, on average, an annual 138 573 to 183 708 lives were saved in the 16 treatment countries, translating to 1.4 to 1.8 million lives saved over a 10-year period.

We carried out a residual regression analysis to better isolate the impact of USG funding by controlling for the confounding effects of non-USG funding during the treatment period. This analysis indicates that [33,34], overall, non-USG funding is not the main driver of our findings. However, additional sensitivity analysis for scenario 2 (*i.e.* considering 15 countries except South Africa as the treatment unit) suggests that non-USG funding contributes between 13% and 28% of our estimated treatment effect. After adjusting for a possible 28% contribution from non-USG donors based on sensitivity tests, we can conservatively estimate that, on average, an annual 99 773 to 132 270 lives were saved in the treatment countries in the absence of confounding from non-USG funding. This translates into about 1.0 to 1.3 million lives saved over 10 years in countries with significant USG funding across a broad range of health areas (and in the absence of non-USG funding). It’s important to clarify that this specific sensitivity analysis does not directly establish a causal effect of USG funding. It suggests a causal impact by ruling out non-USG funding as a potential confounder. These results should be interpreted within the broader context of our quasi-experimental SCA analysis. Our focus is on assessing the sensitivity of our main findings when controlling for non-USG funding, rather than precisely quantifying the direct impact of non-USG funding, which is beyond the scope of this study.

While our study focuses on countries with relatively high USAID funding for practical reasons, such as the broad range of health areas being supported by USAID, it is important to acknowledge the broader context of USG foreign assistance, which includes significant contributions from agencies such as the Centers for Disease Control and Prevention (CDC), the Department of State, *etc*. The relationship between USAID spending and overall USG assistance to LMICs is complex and reciprocal. Our findings should be seen as part of a larger coordinated USG effort. Recognising the contributions from all USG agencies is essential to fully understand the impact of USG foreign assistance on global health. For example, in those countries that included USAID malaria funding and/or funding for HIV/TB, there would also be additional funding for those programs from other US government agencies (CDC, Department of State, US Peace Corps, *etc.*). Therefore, the impact quantified here is better interpreted as representing the impact of USG funding on global health, not only USAID funding. While examining ODA trends by sources (results not shown), we found that non-USG funding per capita is greater than USG funding per capita, on average, in both treatment and control countries, with differences being much larger in treatment countries. The countries in the donor pool that were not included in the synthetic control analysis show a similar pattern. However, we observed a greater rate of growth in USG funding per capita in our treatment countries compared to non-USG donors (although non-USG donors still contribute more ODA in absolute terms). This indicates that USG’s involvement in LMICs not only facilitates direct investments but also leverages resources from the public and private sectors. Additionally, influencing policies and stakeholders, including non-USG donors, may play a role in accelerating mortality reductions among females in the treatment countries, which was reflected in the Theory of Change (**Figure 1**) and supported by our sensitivity analysis (*i.e.* residual regression analysis). The synergy between USG and other donors warrants further review to better understand the underlying mechanisms.

### Why is SCA the appropriate IE method for this?

Given that USG has provided health-related support for a large number of LMICs for several decades, there are a limited number of LMICs to use for comparison that did not receive any support from USG, had similar pre-intervention trends in WRA mortality, and had significant population and data. Some LMICs with limited USG funding (less than six of 20 years of the treatment period) were included in the donor pool (*i.e.* they were eligible to be a comparison) to increase the number of donor countries; this was considered a conservative approach as it would tend to mitigate the treatment effect to some degree under the theory of change.

The SCA can estimate the impact of USG support on WRA mortality because it allows for the comparison to a counterfactual that is constructed via an unbiased process. The SCA methodology is suitable for evaluating population-level interventions where there are a small number of treatment and comparison units (in this case, countries) and does not rely on the assumption of parallel pre-intervention trends in outcomes as do other quasi-experimental methods [38]. Within global health, the methodology has also been used to study the effect of various policy or programmatic initiatives on health and mortality outcomes [39]. When evaluating the impact of global health investments, a critical step involves comparing the outcomes of countries that have received investments with those that have not. However, the uniqueness of each country’s socio-economic, cultural, and health context often means that finding directly comparable control units is inherently challenging. Comparable control units here refer to a group of countries that, in the absence of the health investment under study, would have followed similar trends in health outcomes and determinants as the treatment unit. These units must closely match the treatment unit in key pre-intervention characteristics that could influence the outcome of interest, such as non-USG development assistance, GDP per capita, health system strength, disease prevalence, and socio-political stability.

To circumvent this challenge and construct a valid comparison, we utilised SCA methodology in this paper to quantify the effect of USG global health assistance on WRA mortality. We began by selecting countries receiving six years or less of USAID global health investment during the intervention period but similar enough in observable characteristics to our treatment unit to create a donor pool. The selection criteria for these potential donor countries involved matching pre-treatment indicators relevant to the health outcomes being studied, macroeconomic conditions, non-US development assistance, health indicators, and sociopolitical environments. The synthetic control approach then created a composite unit (the ‘synthetic control’) from this donor pool that, when combined in specific proportions, closely resembled the treated unit’s characteristics before the intervention. This methodology was previously used to assess the impact of USAID funding on under-five mortality [15].

### How do the findings influence global health in terms of accounting for aid?

Analysing the success of USG’s long-term and relatively large investments that cover a broad range of programs such as maternal health, HIV and AIDS, malaria, TB, nutrition, and family planning in reducing preventable deaths among women aged 15-49 has major implications for global health, especially regarding donor accountability and future funding strategies. This positive outcome highlights the power of strategic health funding, making a strong case for sustained or increased support from USAID, other USG agencies, and other major donors. It demonstrates that targeted investments, of robust size and breadth, and under an appropriate theory of change, can yield significant improvements in health, potentially shaping how global health funds allocate resources. These findings can prompt USAID, other USG agencies, and other development partners to reassess funding priorities and ensure resources are directed towards proven interventions and approaches with measurable impacts on reducing mortality. This approach can optimise the global health investment portfolio not only for cost-effectiveness but also for broader, more equitable health outcomes for women and communities worldwide.

### Sensitivity analyses with South Africa and Nigeria

As part of our sensitivity analysis, the SCA model for scenario 2 (excluding South Africa) estimated a slightly larger average effect size of −0.78 for the 2005–2019 period compared to the original SCA. However, placebo tests revealed that the effect estimates remained marginally above the conventional 5% significance level for most of the post-treatment period (Table S4 in the **Online Supplementary Document**). This lack of statistical significance in the treatment effect after excluding South Africa is understandable, given that the country received substantially higher HIV-related USG funding than from non-USG donors [35,36], compared to any other countries in our treatment unit.

On the other hand, our sensitivity analyses clearly indicated that excluding Nigeria from the treatment unit (*i.e.* scenario 3) would result in a larger, statistically significant reduction in WRA mortality compared to the synthetic control in both the SCA and BM. The per capita USAID investment, for example, in Nigeria was relatively low among the treatment unit countries due to the country’s vast population (Figure S2 in the **Online Supplementary Document**), and Nigeria almost did not meet the criteria for inclusion based on funding levels. Nigeria may not have reached a relevant (but unknown) threshold level of per capita funding despite significant total annual global health contributions from USG. In addition, among the countries in the treatment unit, only Nigeria showed a secular, plateaued trend in WRA mortality rate during the study period that also suggests investments in health (donor plus domestic) may have been insufficient, at least on a per capita basis. The persistent high mortality among WRA in Nigeria may also be attributed to a complex interplay of factors. Women’s perceptions and experiences with the quality of health care significantly influence their use of health services [40,41], often favouring traditional over formal health care settings due to perceived better care and cultural suitability [42,43]. In northern Nigeria, cultural practices such as female seclusion discourage the use of external health services during childbirth [42]. Additionally, issues with the performance of community health workers, such as inadequate training, poor working conditions, and suboptimal postnatal services, exacerbate the situation [40,44]. Addressing these multifaceted issues, in addition to robust health investment, requires a tailored strategy that includes better understanding and improving women’s health care experiences and perceptions.

### Strengths

Recent methodological advancements have addressed many of the limitations of the traditional SCA approach, with new methods demonstrating improved predictive performance, model robustness, relaxed identification assumptions, and interpretable uncertainty intervals. We utilised both the SCA and a novel Bayesian dynamic multilevel latent factor model and found similar results that support the hypothesis that increased USG investments in global health are associated with decreases in the mortality rate for WRA.

### Limitations

While the evidence suggests a positive impact of relatively large and broad USG global health investments on WRA mortality, the study has several limitations. A limitation of the original SCA method is its inability to produce uncertainty intervals, which is desirable for making statistical inferences as units are not sampled probabilistically – assumptions of linearity for unobserved covariates and additive effects of USG funding compound this issue. Therefore, we included the BM, placebo tests, and multiple sensitivity analyses to increase the robustness of the estimates for treatment effects. There are also inherent limitations in our donor pool of countries. For example, no country can perfectly mimic the HIV epidemic in Southern or Eastern Africa, so we did the best we could to create a synthetic control using the available donor countries. Though the HIV prevalence among the population ages 15–49 between the treatment unit and synthetic control was not vastly different (Table S2 in the **Online Supplementary Document**), our sensitivity analysis for scenario 3 (after excluding South Africa) revealed a larger, statistically significant effect size during the post-treatment period. Additionally, the outcome variable of WRA mortality may not be sufficiently sensitive to change in some cases or may experience lags in changes over time [45], and thus, the effect sizes may not appropriately reflect in the findings of our analysis. The classification of the treatment year beginning in 2005 is also a study design choice and could easily have been earlier or later. However, we ran sensitivity analyses reinforcing the decision to use 2005 as the treatment year. Furthermore, there was missing funding data for 2005–2006, which may have impacted the accuracy of the analyses. To further investigate this, we carried out additional sensitivity analysis using different treatment years and found support for using 2005 as the treatment year for analysis (Text S5 in the **Online Supplementary Document**). Lastly, among USG agencies, we only had fully detailed funding data from USAID, and while these data may be a useful proxy for the full USG funding support, and while our residual sensitivity analysis supports the inference of the important role of USG global health funding in mortality reductions among WRA, disaggregating between the contributions of different agencies is not possible with current data.

## CONCLUSIONS

While some studies have explored the impact of donor funding on under-five, maternal, and adult mortality, to the best of our knowledge, this is the first study to look at WRA mortality. Well-resourced USG global health investments have contributed to declines in mortality among WRA during the study period, resulting in an estimated 1.0–1.3 million lives saved over a ten-year period. Contributing to programs that together combat a broad range of health areas, such as HIV and AIDS, maternal and child health, nutrition, family planning, and other infectious diseases and health systems strengthening, has a direct impact on WRA at the country level. The synergistic effect of USG multi-pronged strategies, as documented in numerous studies [46–49], in partnership with country governments, appears to have played a crucial role in reducing WRA mortality, most likely exceeding the impact of a more narrow focus on single health conditions or areas alone. This holistic approach outlined in the Theory of Change, encompassing access to comprehensive health care, promotion of healthy behaviours, system strengthening, leveraging other partners’ investments, policy influence, and program evaluation, creates a robust framework that addresses the broad spectrum of factors affecting women’s health during their reproductive years. By interlinking these elements, the combined impact significantly enhances health outcomes for WRA, demonstrating the benefit of a coordinated, holistic health strategy over isolated interventions for this population. By working closely with countries to continue to improve access to coverage and quality high-impact interventions, substantial improvements in the health of vulnerable populations are possible and measurable.

## Additional material


Online Supplementary Document
