# Supplementary material for: Accounting for aid: estimating the impact of United States’ global health investments on mortality among women of reproductive age using synthetic control and Bayesian methods
Source: J Glob Health. 2025 Mar 21;15:04067. doi: 10.7189/jogh.15.04067 (PMC11927036; doi:10.7189/jogh.15.04067)
Supplement: Online Supplementary Document [file jogh-15-04067-s001.pdf]

Supplement to: Ahsan KZ, Angeles G, Choudhury A, Singh K, Taylor TM, Majid F, Lucas R, Cohen RL, Gawande AA, Weiss W. Accounting for aid: estimating the impact of United States' global health investments on mortality among women of reproductive age using synthetic control and Bayesian methods. J Glob Health. 2025;15:04067.

Title: Accounting for aid: estimating the impact of United States' global health investments on mortality among women of reproductive age using synthetic control and Bayesian methods

Authors: Karar Zunaid Ahsan[1,2]*; Gustavo Angeles[3]; Allysha Choudhury[3]; Kavita Singh[3]; Tory M Taylor[2]; Farhan Majid[4]; Rachel Lucas[5]; Robert L Cohen[4]; Atul A Gawande[6]; William Weiss[7]

Affiliations:

[1] Department of Public Health Leadership and Practice, Gillings School of Global Public Health, University of North Carolina at Chapel Hill, Chapel Hill, North Carolina, USA

[2] Data for Impact (D4I), Carolina Population Center, University of North Carolina at Chapel Hill, Chapel Hill, North Carolina, USA

[3] Department of Maternal and Child Health, Gillings School of Global Public Health, University of North Carolina at Chapel Hill, Chapel Hill, North Carolina, USA

[4] Public Health Institute, Oakland, California, USA

[5] Office of the Assistant Administrator, Bureau for Global Health, U.S. Agency for International Development, Washington, DC, USA

[6] Ariadne Labs at Brigham & Women's Hospital and Harvard TH Chan School of Public Health, Boston, Massachusetts, USA

[7] Department of International Health, Johns Hopkins University, Baltimore, Maryland, USA

* Correspondence to: Dr. Karar Zunaid Ahsan,

Text S1. Additional information on Bayesian Dynamic Multilevel Latent Factor Model

The Bayesian dynamic multilevel latent factor model (BM) adopts a Bayesian causal inference framework to create a counterfactual to treatment units and estimate treatment effects with uncertainty intervals (Pang et al., 2021). It incorporates multiple sources of heterogeneity and dynamics present in the data by accommodating flexible functional forms, using latent factor terms, and allowing covariate coefficients to vary by unit or over time. Bayesian shrinkage priors conduct variable and factor selection, while a Markov Chain Monte Carlo algorithm is used to facilitate model and parameter estimation. Bayesian prediction generates a posterior distribution of counterfactual outcomes along with 90% credible intervals, which span the 5% and 95% quantiles of the posterior distribution and are acceptable for usage in Bayesian modeling (MacElreath, 2020).

The BM method requires a large number of pre-treatment periods and a greater number of donor units than treatment units in order to accurately estimate the treatment effect. Assumptions for the BM include the following: a cross-sectional stable unit treatment value (treatment and control groups are independent and not influenced by each other); no anticipation effects; individualistic assignment and positivity (the countries receiving USAID global health investments did not depend on the potential outcomes of funding for other countries); latent ignorability (conditional on latent variables, whether a country received funding from USAID is independent of any missing or observed untreated outcomes for that country); and, feasible data extraction (unobserved factors of each country can be approximated by lower-rank matrices). Both the limited and expanded predictor set BM resulted in the identification of three-factor terms, suggesting that there are three underlying latent variables that summarized the patterns and distributions of the predictor variables included in the models.

Figure S1. Total USAID funding for global health programs in 101 countries, 1990–2019

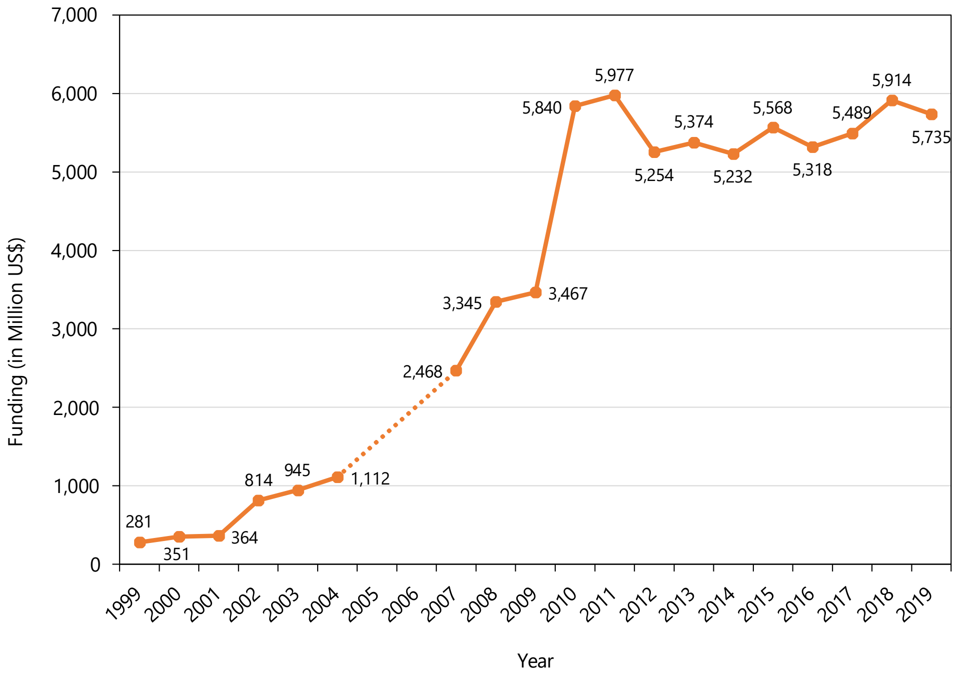

Figure S2. Selection of treatment unit based on total and per-capita USAID funding

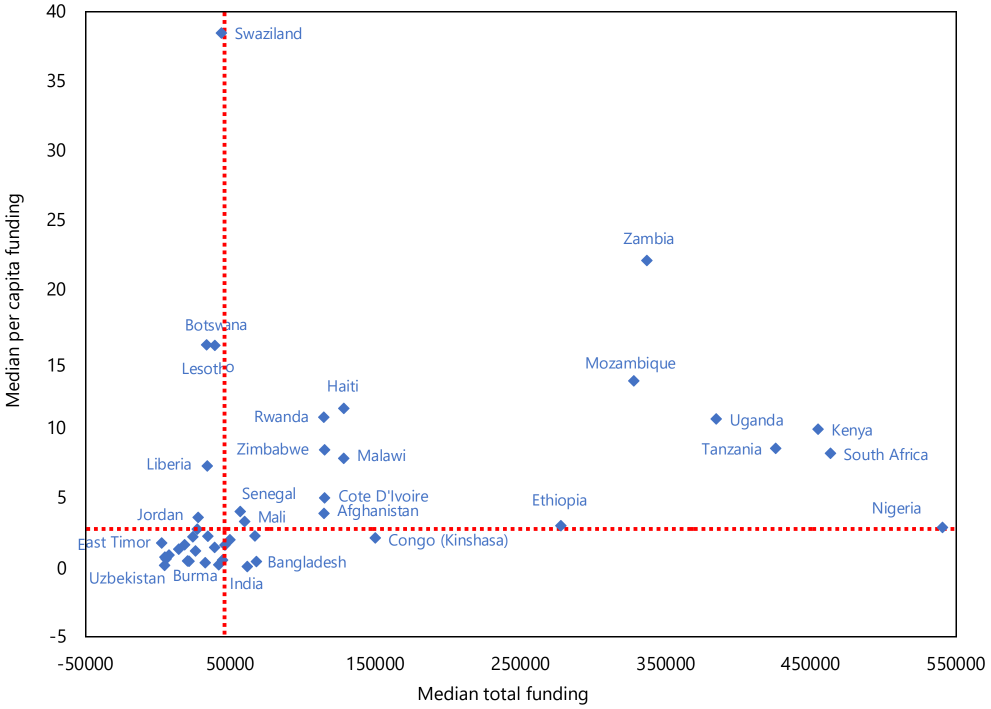

Figure S3. Trends in mortality rate among women of reproductive age in the treatment and donor countries, 1990–2019

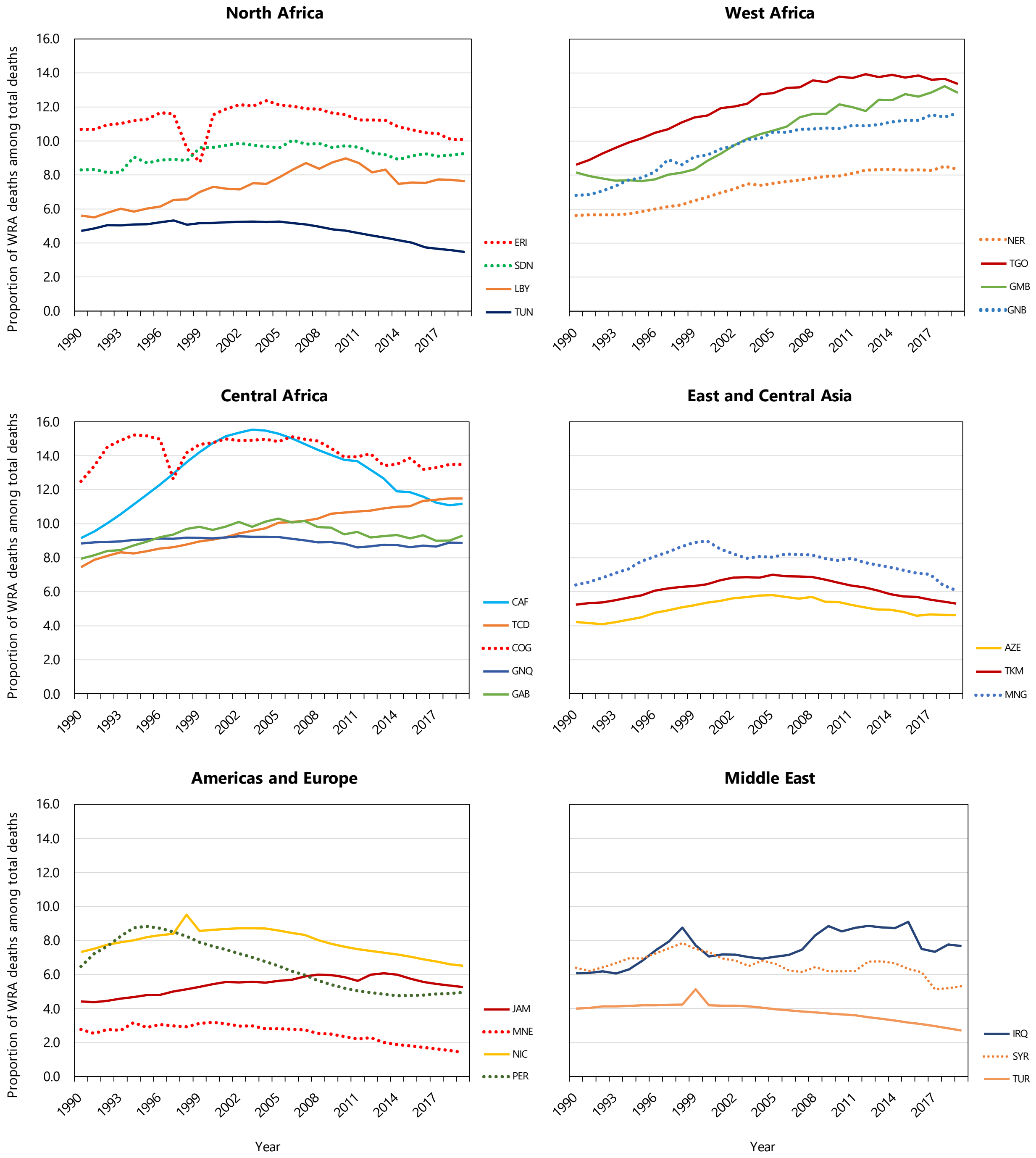

Note: TUR: Turkey; TUN: Tunisia; TKM: Turkmenistan; MNE: Montenegro; IRQ: Iraq; SYR: Syrian Arab Republic; MNG: Mongolia; LBY: Libya; GNB: Guinea-Bissau; GMB: Gambia; GAB: Gabon; GNQ: Equatorial Guinea; COG: Congo (Brazzaville); TCD: Chad; TGO: Togo; SDN: Sudan; NER: Niger; PER: Peru; NIC: Nicaragua; JAM: Jamaica; ERI: Eritrea; CAF: Central African Republic; AZE: Azerbaijan.

Figure S4. Trends in mortality rate among WRA in the treatment and donor units, 1990–2019

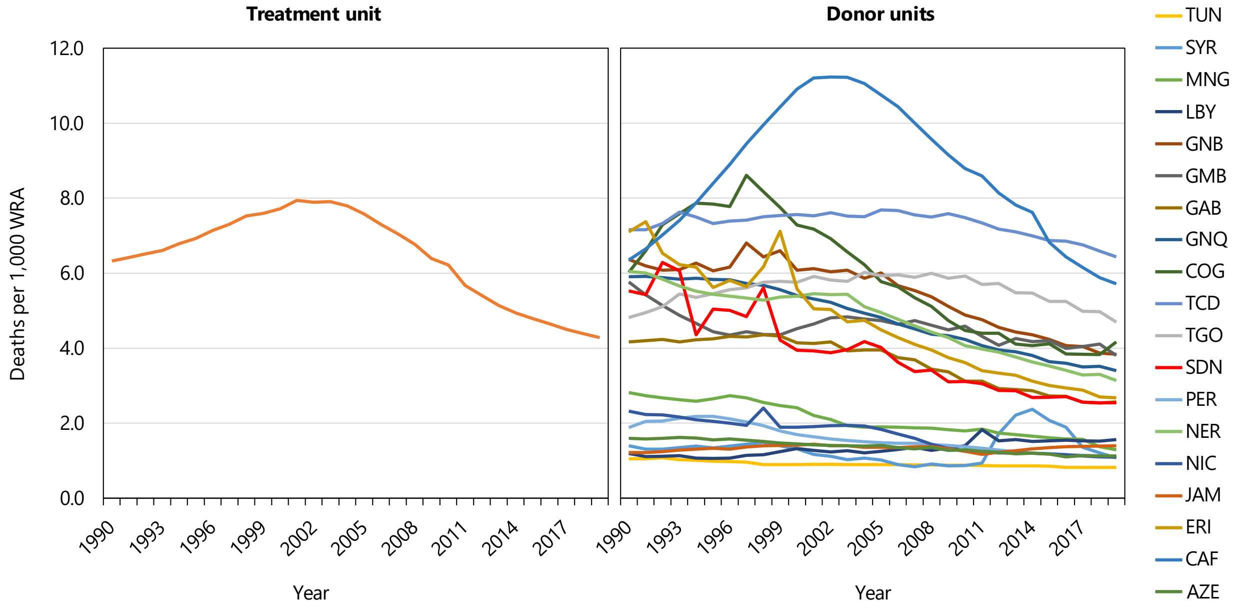

Note: TUN: Tunisia; SYR: Syrian Arab Republic; MNG: Mongolia; LBY: Libya; GNB: Guinea-Bissau; GMB: Gambia; GAB: Gabon; GNQ: Equatorial Guinea; COG: Congo (Brazzaville); TCD: Chad; TGO: Togo; SDN: Sudan; NER: Niger; PER: Peru; NIC: Nicaragua; JAM: Jamaica; ERI: Eritrea; CAF: Central African Republic; AZE: Azerbaijan.

Figure S5. Countries selected as treated (n=16) and donor (n=19) units

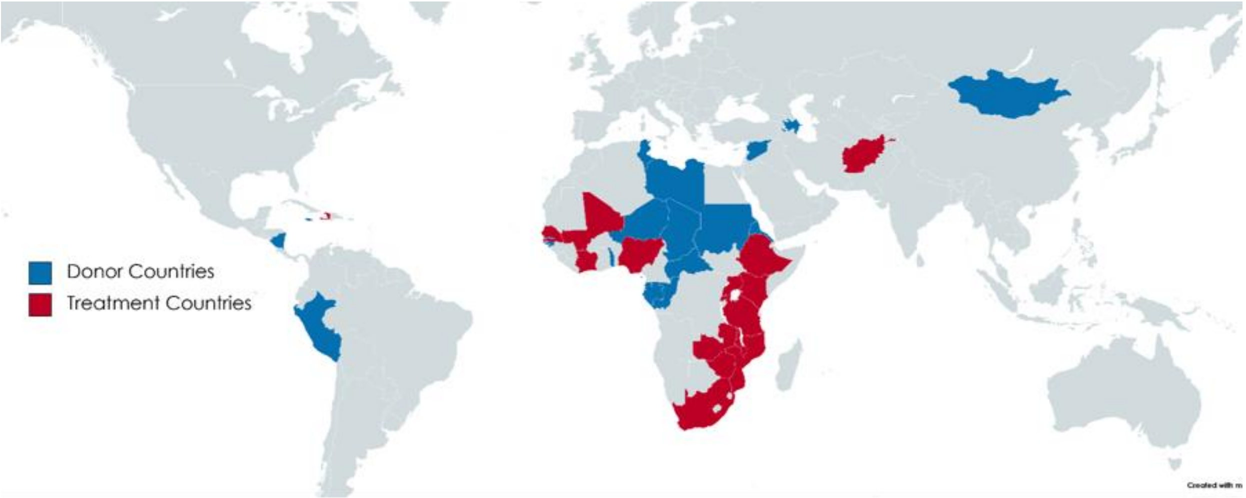

Text S2. Country selection process for donor pool

	FUNDING					DATA AVAILABILITY FOR PREDICTORS												
**LOW- AND -MIDDLE INCOME COUNTRIES IN 1999/2000**	Funding received during 2007-2019	Funding received during 1999-2004 only	No funding received 1999-2019	**POPULATION SIZE >500,000 IN 2004**	**ASCENDING TREND IN % WRA DEATHS 1990-2004**	HIV prevalence, total (% of 15–49)	Tuberculosis case detection rate	Contraceptive prevalence rate (any method)	Immunization coverage, measles	Net non-USG ODA received per capita	Mean years of schooling for females	% urban population	Total fertility rate	Total population (logged)	Female labor force participation	GDP per capita	Polity5 score	**COUNTRY CREATED BEFORE 2005**
Albania																		
Algeria																		
American Samoa																		
Argentina																		
Azerbaijan																		
Barbados & East. Caribbean																		
Belarus																		
Belize																		
Bhutan																		
Bolivia																		
Bosnia and Herzegovina																		
Bulgaria																		
Cabo Verde																		
Central African Republic																		
Chad																		
Colombia																		
Comoros																		
Congo																		
Costa Rica																		
Croatia																		
Cuba																		
Dominica																		
Ecuador																		
El Salvador																		
Equatorial Guinea																		
Eritrea																		
Fiji																		
Gabon																		
Gambia																		
Grenada																		

Guinea-Bissau	No	No	Yes	Yes	Yes	Yes	Yes	Yes	Yes	Yes	Yes	Yes	Yes	Yes	Yes	Yes	Yes	Yes
Iran	No	No	Yes	Yes	No	Not applicable	Not applicable	Not applicable	Not applicable	Not applicable	Not applicable	Not applicable	Not applicable	Not applicable	Not applicable	Not applicable	Not applicable	Not applicable
Iraq	Yes	No	No	Yes	Yes	No	Yes	Yes	Yes	Yes	Yes	Yes	Yes	Yes	Yes	Yes	Yes	Not applicable
Jamaica	Yes	No	No	Yes	Yes	Yes	Yes	Yes	Yes	Yes	Yes	Yes	Yes	Yes	Yes	Yes	Yes	Yes
Kiribati	No	No	Yes	No	Not applicable	Not applicable	Not applicable	Not applicable	Not applicable	Not applicable	Not applicable	Not applicable	Not applicable	Not applicable	Not applicable	Not applicable	Not applicable	Not applicable
Kosovo	Yes	No	No	Yes	No	Not applicable	Not applicable	Not applicable	Not applicable	Not applicable	Not applicable	Not applicable	Not applicable	Not applicable	Not applicable	Not applicable	Not applicable	Not applicable
Lebanon	No	No	Yes	Yes	No	Not applicable	Not applicable	Not applicable	Not applicable	Not applicable	Not applicable	Not applicable	Not applicable	Not applicable	Not applicable	Not applicable	Not applicable	Not applicable
Libya	No	No	Yes	Yes	Yes	Yes	Yes	Yes	Yes	Yes	Yes	Yes	Yes	Yes	Yes	Yes	Yes	Yes
Macedonia	No	Yes	No	Yes	No	Not applicable	Not applicable	Not applicable	Not applicable	Not applicable	Not applicable	Not applicable	Not applicable	Not applicable	Not applicable	Not applicable	Not applicable	Not applicable
Malaysia	No	No	Yes	Yes	No	Not applicable	Not applicable	Not applicable	Not applicable	Not applicable	Not applicable	Not applicable	Not applicable	Not applicable	Not applicable	Not applicable	Not applicable	Not applicable
Maldives	No	No	Yes	No	Not applicable	Not applicable	Not applicable	Not applicable	Not applicable	Not applicable	Not applicable	Not applicable	Not applicable	Not applicable	Not applicable	Not applicable	Not applicable	Not applicable
Marshall Island	No	No	Yes	No	Not applicable	Not applicable	Not applicable	Not applicable	Not applicable	Not applicable	Not applicable	Not applicable	Not applicable	Not applicable	Not applicable	Not applicable	Not applicable	Not applicable
Mauritania	No	No	Yes	Yes	No	Not applicable	Not applicable	Not applicable	Not applicable	Not applicable	Not applicable	Not applicable	Not applicable	Not applicable	Not applicable	Not applicable	Not applicable	Not applicable
Mauritius	No	No	Yes	Yes	No	Not applicable	Not applicable	Not applicable	Not applicable	Not applicable	Not applicable	Not applicable	Not applicable	Not applicable	Not applicable	Not applicable	Not applicable	Not applicable
Mexico	Yes	No	No	Yes	No	Not applicable	Not applicable	Not applicable	Not applicable	Not applicable	Not applicable	Not applicable	Not applicable	Not applicable	Not applicable	Not applicable	Not applicable	Not applicable
Micronesia	No	No	Yes	No	Not applicable	Not applicable	Not applicable	Not applicable	Not applicable	Not applicable	Not applicable	Not applicable	Not applicable	Not applicable	Not applicable	Not applicable	Not applicable	Not applicable
Moldova	No	Yes	No	Yes	No	Not applicable	Not applicable	Not applicable	Not applicable	Not applicable	Not applicable	Not applicable	Not applicable	Not applicable	Not applicable	Not applicable	Not applicable	Not applicable
Mongolia	No	No	Yes	Yes	Yes	Yes	Yes	Yes	Yes	Yes	Yes	Yes	Yes	Yes	Yes	Yes	Yes	Yes
Montenegro	No	Yes	No	Yes	Yes	Yes	Yes	Yes	Yes	Yes	Yes	Yes	Yes	Yes	Yes	Yes	Yes	No
Morocco	No	Yes	No	Yes	No	Not applicable	Not applicable	Not applicable	Not applicable	Not applicable	Not applicable	Not applicable	Not applicable	Not applicable	Not applicable	Not applicable	Not applicable	Not applicable
North Korea	No	No	Yes	Yes	No	Not applicable	Not applicable	Not applicable	Not applicable	Not applicable	Not applicable	Not applicable	Not applicable	Not applicable	Not applicable	Not applicable	Not applicable	Not applicable
Nicaragua	Yes	No	No	Yes	Yes	Yes	Yes	Yes	Yes	Yes	Yes	Yes	Yes	Yes	Yes	Yes	Yes	Yes
Niger	Yes	No	No	Yes	Yes	Yes	Yes	Yes	Yes	Yes	Yes	Yes	Yes	Yes	Yes	Yes	Yes	Yes
Palau	No	No	Yes	No	Not applicable	Not applicable	Not applicable	Not applicable	Not applicable	Not applicable	Not applicable	Not applicable	Not applicable	Not applicable	Not applicable	Not applicable	Not applicable	Not applicable
Panama	Yes	No	No	Yes	No	Not applicable	Not applicable	Not applicable	Not applicable	Not applicable	Not applicable	Not applicable	Not applicable	Not applicable	Not applicable	Not applicable	Not applicable	Not applicable
Paraguay	Yes	No	No	Yes	No	Not applicable	Not applicable	Not applicable	Not applicable	Not applicable	Not applicable	Not applicable	Not applicable	Not applicable	Not applicable	Not applicable	Not applicable	Not applicable
Peru	Yes	No	No	Yes	Yes	Yes	Yes	Yes	Yes	Yes	Yes	Yes	Yes	Yes	Yes	Yes	Yes	Yes
Romania	No	Yes	No	Yes	No	Not applicable	Not applicable	Not applicable	Not applicable	Not applicable	Not applicable	Not applicable	Not applicable	Not applicable	Not applicable	Not applicable	Not applicable	Not applicable
Russia	Yes	No	No	Yes	No	Not applicable	Not applicable	Not applicable	Not applicable	Not applicable	Not applicable	Not applicable	Not applicable	Not applicable	Not applicable	Not applicable	Not applicable	Not applicable
Saint Lucia	No	No	Yes	No	Not applicable	Not applicable	Not applicable	Not applicable	Not applicable	Not applicable	Not applicable	Not applicable	Not applicable	Not applicable	Not applicable	Not applicable	Not applicable	Not applicable
Saint Vincent & Grenadines	No	No	Yes	No	Not applicable	Not applicable	Not applicable	Not applicable	Not applicable	Not applicable	Not applicable	Not applicable	Not applicable	Not applicable	Not applicable	Not applicable	Not applicable	Not applicable
Samoa	No	No	Yes	No	Not applicable	Not applicable	Not applicable	Not applicable	Not applicable	Not applicable	Not applicable	Not applicable	Not applicable	Not applicable	Not applicable	Not applicable	Not applicable	Not applicable
Sao Tome & Principe	Yes	No	No	No	Not applicable	Not applicable	Not applicable	Not applicable	Not applicable	Not applicable	Not applicable	Not applicable	Not applicable	Not applicable	Not applicable	Not applicable	Not applicable	Not applicable
Serbia	No	Yes	No	Yes	No	Not applicable	Not applicable	Not applicable	Not applicable	Not applicable	Not applicable	Not applicable	Not applicable	Not applicable	Not applicable	Not applicable	Not applicable	Not applicable
Solomon Island	No	No	Yes	No	Not applicable	Not applicable	Not applicable	Not applicable	Not applicable	Not applicable	Not applicable	Not applicable	Not applicable	Not applicable	Not applicable	Not applicable	Not applicable	Not applicable
Sri Lanka	No	Yes	No	Yes	No	Not applicable	Not applicable	Not applicable	Not applicable	Not applicable	Not applicable	Not applicable	Not applicable	Not applicable	Not applicable	Not applicable	Not applicable	Not applicable
Sudan	Yes	No	No	Yes	Yes	Yes	Yes	Yes	Yes	Yes	Yes	Yes	Yes	Yes	Yes	Yes	Yes	Yes
Suriname	No	No	Yes	Yes	No	Not applicable	Not applicable	Not applicable	Not applicable	Not applicable	Not applicable	Not applicable	Not applicable	Not applicable	Not applicable	Not applicable	Not applicable	Not applicable
Syrian Arab Republic	No	No	Yes	Yes	Yes	Yes	Yes	Yes	Yes	Yes	Yes	Yes	Yes	Yes	Yes	Yes	Yes	Yes
Thailand	Yes	No	No	Yes	No	Not applicable	Not applicable	Not applicable	Not applicable	Not applicable	Not applicable	Not applicable	Not applicable	Not applicable	Not applicable	Not applicable	Not applicable	Not applicable
Togo	Yes	No	No	Yes	Yes	Yes	Yes	Yes	Yes	Yes	Yes	Yes	Yes	Yes	Yes	Yes	Yes	Yes
Tonga	No	No	Yes	No	Not applicable	Not applicable	Not applicable	Not applicable	Not applicable	Not applicable	Not applicable	Not applicable	Not applicable	Not applicable	Not applicable	Not applicable	Not applicable	Not applicable
Tunisia	No	No	Yes	Yes	Yes	Yes	Yes	Yes	Yes	Yes	Yes	Yes	Yes	Yes	Yes	Yes	Yes	Yes
Turkey	No	No	Yes	Yes	Yes	No	Yes	Yes	Yes	Yes	Yes	Yes	Yes	Yes	Yes	Yes	Yes	Not applicable
Turkmenistan	Yes	No	No	Yes	Yes	No	Yes	Yes	Yes	Yes	Yes	Yes	Yes	Yes	Yes	Yes	Yes	Not applicable
Tuvalu	No	No	Yes	No	Not applicable	Not applicable	Not applicable	Not applicable	Not applicable	Not applicable	Not applicable	Not applicable	Not applicable	Not applicable	Not applicable	Not applicable	Not applicable	Not applicable
Venezuela	Yes	No	No	Yes	No	Not applicable	Not applicable	Not applicable	Not applicable	Not applicable	Not applicable	Not applicable	Not applicable	Not applicable	Not applicable	Not applicable	Not applicable	Not applicable
**Number of countries**	**77**			**59**	**23**	**20**												**19**

Yes No Not applicable

Table S1. Definition of study variables

No.	Indicator	Definition	Data source
1.	HIV prevalence, total (% of population ages 15–49)	The percentage of people ages 15-49 who are infected with HIV.	WDI via UNAIDS estimates
2.	Tuberculosis case detection rate	The number of new and relapse tuberculosis cases notified to WHO in a given year, divided by WHO's estimate of the number of incident tuberculosis cases for the same year, expressed as a percentage. Estimates for all years are recalculated as new information becomes available and techniques are refined, so they may differ from those published previously.	WDI via World Health Organization's Global Tuberculosis Report.
3.	Contraceptive prevalence rate (any method)	The percentage of married women ages 15-49 who are practicing, or whose sexual partners are practicing, any method of contraception (modern or traditional). Modern methods of contraception include female and male sterilization, oral hormonal pills, the intra-uterine device (IUD), the male condom, injectables, the implant (including Norplant), vaginal barrier methods, the female condom, and emergency contraception. Traditional methods of contraception include rhythm (e.g., fertility awareness-based methods, periodic abstinence), withdrawal, and other traditional methods.	WDI/Alkema et al. (2013)<br><br>Household surveys, including Demographic and Health Surveys and Multiple Indicator Cluster Surveys, compiled by United Nations Population Division.
4.	Immunization coverage, measles	The percentage of children ages 12-23 months who received the measles vaccination before 12 months or at any time before the survey. A child is considered adequately immunized against measles after receiving one dose of vaccine.	WDI via WHO and UNICEF (who.int/immunization/monitoring_surveillance/en)

5.	Net ODA received from non-USG countries per capita (current US$)	The total net Official Development Assistance Disbursements per capita received from all DAC countries, excluding the United States. This includes disbursements from both bilateral and multilateral funds from DAC countries to recipient countries.	Organization for Economic Cooperation and Development Database (OECD Data Explorer)
6.	Mean years of schooling for females	Average number of completed years of education of a country's female population aged 25 years and older, excluding years spent repeating individual grades.	UNDP Human Development Report, compiled from Barro and Lee (2018), ICF Macro Demographic and Health Surveys (various years), OECD (2022), UNESCO Institute for Statistics (2022), and UNICEF Multiple Indicator Cluster Surveys (various years).
7.	% urban population	Proportion of people living in urban areas as defined by national statistical offices. The data are collected and smoothed by the United Nations Population Division.	WDI via United Nations Population Division's World Urbanization Prospects: 2018 Revision.
8.	Total fertility rate	The number of children that would be born to a woman if she were to live to the end of her childbearing years and bear children in accordance with age-specific fertility rates of the specified year.	WDI via United Nations Population Division's World Population Prospects: 2022 Revision; census reports and other statistical publications from national statistical offices; Eurostat: Demographic Statistics; United Nations Statistical Division's Population and Vital Statistics Report (various years); U.S. Census Bureau: International Database; and Secretariat of the Pacific Community: Statistics and Demography Programme.
9.	Total population (logged)	Midyear estimates of the de facto definition of population, which counts all residents regardless of legal status or citizenship.	WDI via United Nations Population Division's World Population Prospects: 2022 Revision; census reports and other statistical publications from national statistical offices; Eurostat: Demographic Statistics; United Nations Statistical Division's Population and Vital Statistics Report (various years); U.S. Census Bureau: International Database; and Secretariat of the Pacific Community: Statistics and Demography Programme.

10.	Female labor force participation	The percentage of the total shows the extent to which women are active in the labor force. Labor force comprises people ages 15 and older who supply labor for producing goods and services during a specified period.	WDI estimates are based on data obtained from International Labour Organization and United Nations Population Division.
11.	GDP per capita	Gross domestic product divided by midyear population. GDP is the sum of gross value added by all resident producers in the economy plus any product taxes and minus any subsidies not included in the value of the products. It is calculated without making deductions for depreciation of fabricated assets or for depletion and degradation of natural resources. Data are in current U.S. dollars.	WDI via World Bank national accounts data, and OECD National Accounts data files.
12.	Polity5 score	The "Polity Score" captures this regime authority spectrum on a 21-point scale ranging from -10 (hereditary monarchy) to +10 (consolidated democracy). The Polity scores can also be converted into regime categories in a suggested three-part categorization of "autocracies" (-10 to -6), "anocracies" (-5 to +5 and three special values: -66, -77 and -88), and "democracies" (+6 to +10).	Integrated Network for Societal Conflict Research (INSCR) in the Center for Systemic Peace (https://www.systemicpeace.org/index.html)

Notes: WDI: World Development Indicators (https://data.worldbank.org/); Alkema et al. (2013): DOI: 10.1016/S0140-6736(12)62204-1; Barro-Lee (2018): Barro-Lee Educational Attainment Dataset (http://barrolee.com/); OECD Data Explorer (https://data-explorer.oecd.org/)

Table S2. Predictor means between the treatment unit and synthetic control for the optimized synthetic control analysis model

Predictors	Treatment unit	Synthetic control
Net non-USG ODA received per capita (current USD)	18.37	36.57
Tuberculosis case detection rate	34.73	40.24
Contraceptive prevalence rate (any method)	17.23	11.91
Immunization coverage, measles	53.69	48.49
% urban population	27.73	34.95
Total fertility rate	6.01	6.31
Total population (logged)	17.87	15.00
HIV prevalence, total (% of population ages 15–49)	4.84	3.66
Female labour force participation	48.43	46.55
GDP per capita (current USD)	614.08	600.23
Polity5 score	(0.55)	(0.70)
Mean years of schooling for females	2.67	1.51
WRA Mortality Rate 2004	7.80	7.80
WRA Mortality Rate 1997	7.31	7.30
WRA Mortality Rate 1990	6.32	6.30

Notes: USG: The United States government; ODA: overseas development assistance; USD: United States dollar; HIV: human immunodeficiency virus; GDP: gross domestic product; Polity5: policy score by INSCR; WRA: women of reproductive age

Figure S6. Placebo testing (treatment unit and 19 placebos) for the optimized synthetic control analysis model

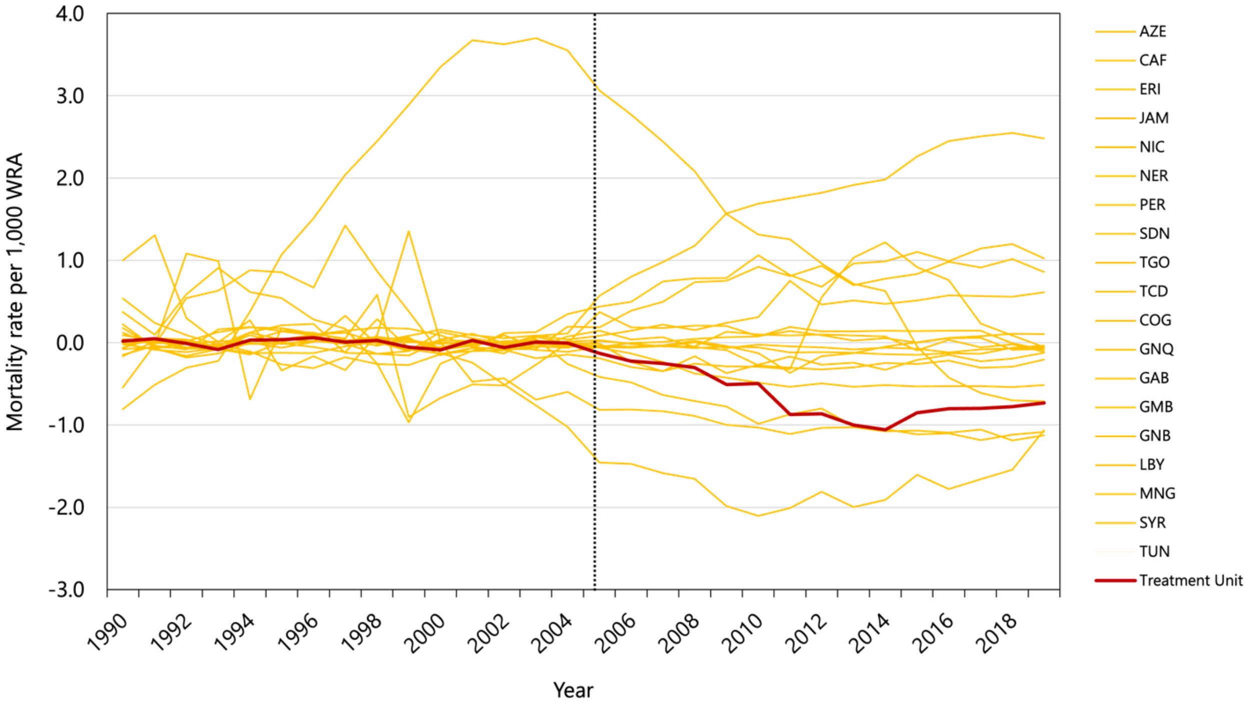

Note: AZE: Azerbaijan; CAF: Central African Republic; ERI: Eritrea; JAM: Jamaica; NIC: Nicaragua; PER: Peru; NER: Niger; SDN: Sudan; TGO: Togo; TCD: Chad; COG: Congo (Brazzaville); GNQ: Equatorial Guinea; GAB: Gabon; GMB: Gambia; GNB: Guinea-Bissau; LBY: Libya; MNG: Mongolia; SYR: Syrian Arab Republic; Tun: Tunisia

Figure S7. Bayesian model inference of treatment effects by individual country with 90% credible interval (limited and expanded predictor set)

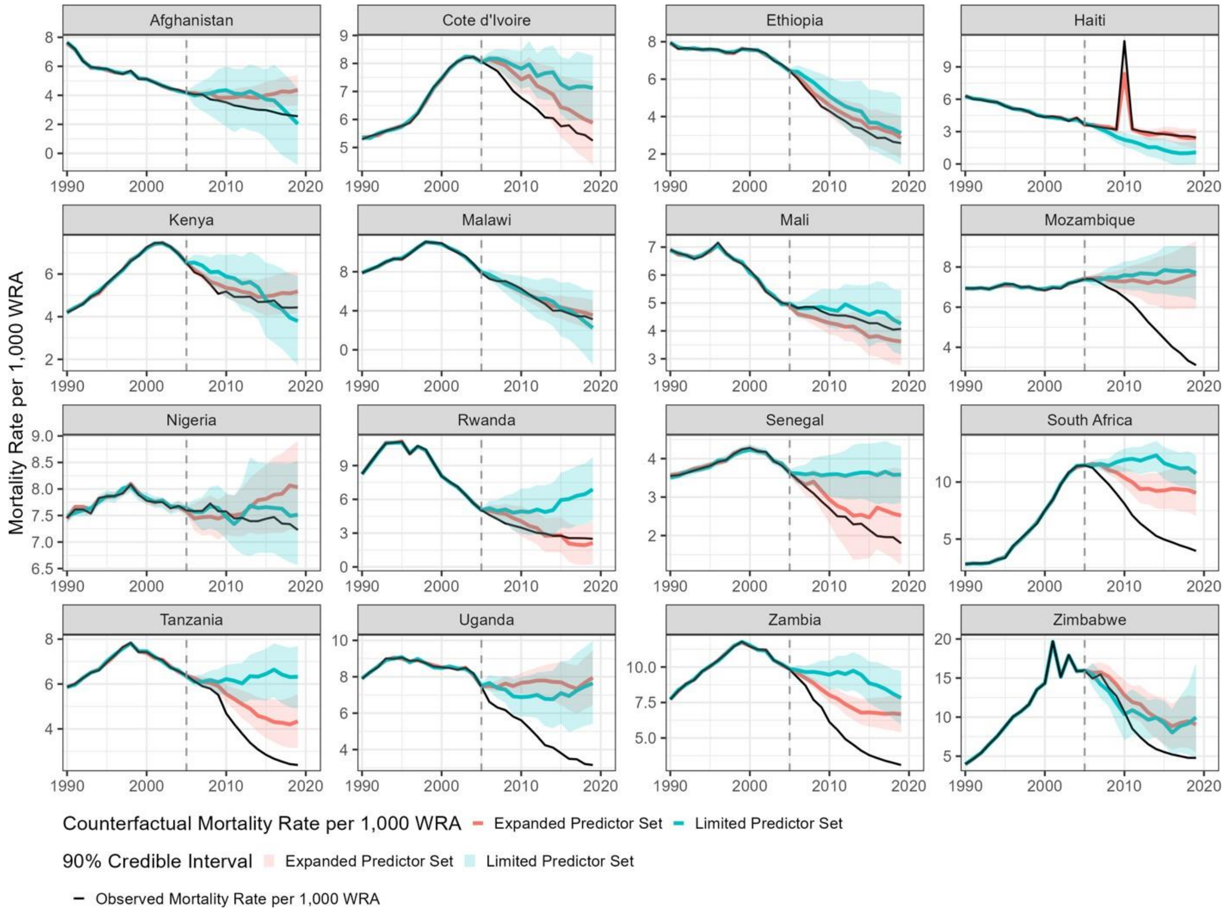

Figure S8. Estimated effect sizes in the optimized synthetic control analysis model and sensitivity analyses (i.e., Scenario 2 and Scenario 3)

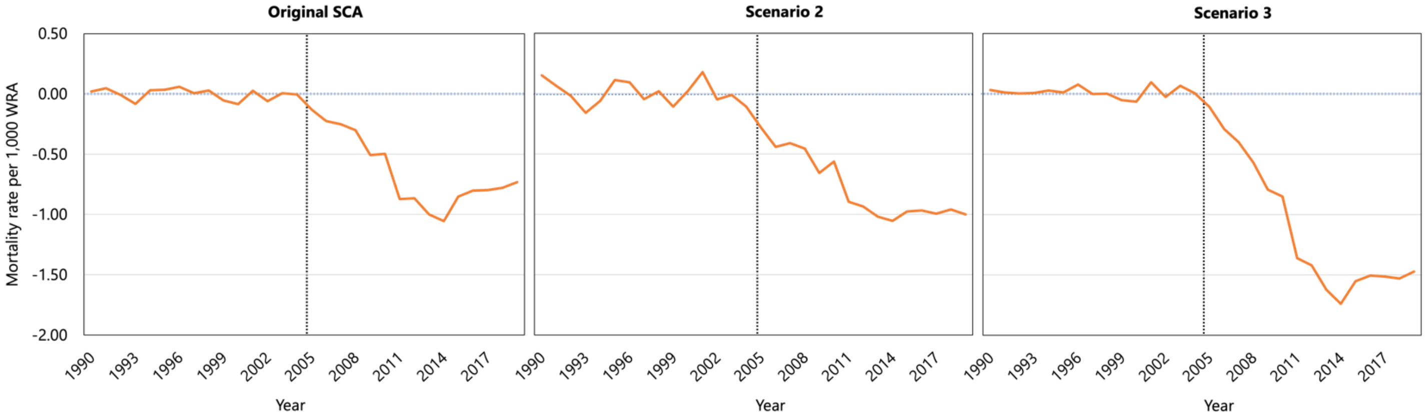

Text S3. Findings from sensitivity analyses

For sensitivity analysis, we carried out traditional SCAs by dropping South Africa (Scenario 2) and Nigeria (Scenario 3) from the treatment unit and compared the findings with our original SCA (Scenario 1).

The rationale for dropping South Africa from the treatment unit was that its cause of death and burden of diseases were highly skewed toward HIV and AIDS, and it received substantial HIV-related funding from USG sources (viz., PEPFAR) compared to non-USG donors, unlike the other countries in our treatment unit. We also ran a sensitivity analysis by dropping Nigeria because, among the countries in the treatment unit, only Nigeria showed an increasing share of women of reproductive age (WRA) deaths among all deaths and a secular, plateaued trend in WRA mortality rate during a substantial part of the study period (see Figure S9).

Figure S9. Trends in WRA mortality in Nigeria, 1990–2019

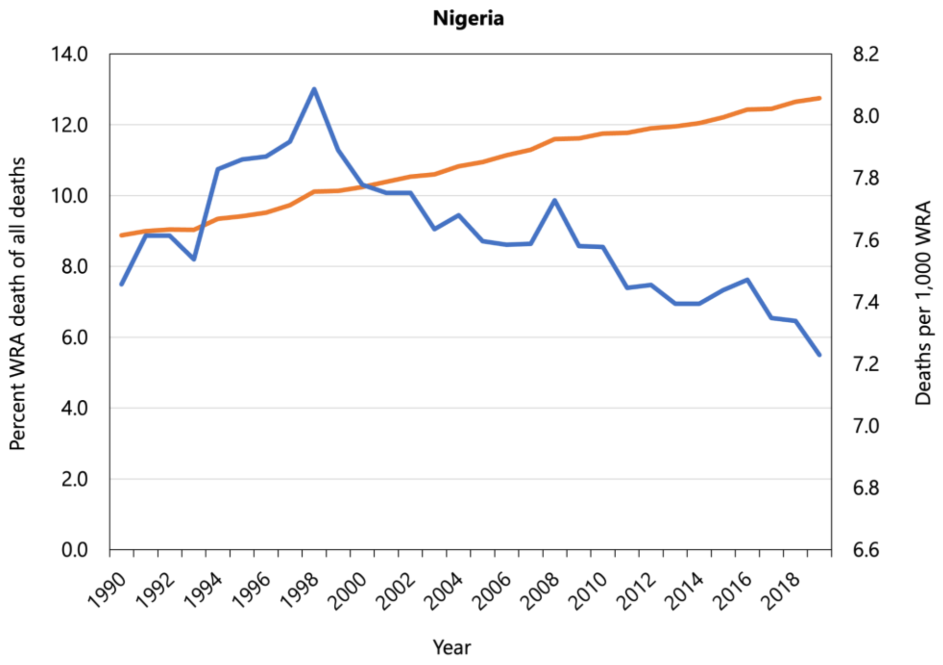

Our SCA model's fit for scenario 2 (i.e., without South Africa) was good (RMSPE = 0.0964072), as seen in Figure S5-2. The resulting synthetic control was a weighted average of the outcomes in Chad (47.4%), Congo (17.7%), the Central African Republic (17.3%), Eritrea (8.9%), Equatorial Guinea (8.7%), and Niger (0.001%). Overall, the comparison of predictors used for SCA indicated a good resemblance of pre-intervention predictors between the treatment unit and its synthetic control (see Table S3). During the post-intervention period, the mortality rate among WRA in the treatment group of countries decreased faster than the synthetic control (see Figure S10). The placebo test shows that the effect estimates remain just outside the conventional significance level of 5% for most of the post-treatment period (see Table S4).

Table S3. Predictor means between the treatment unit (without South Africa) and synthetic control

**Predictors**	**Treatment unit w/o South Africa**	**Synthetic control**
Net non-USG ODA received (current $US) per capita	19.18	35.26
Tuberculosis case detection rate	33.48	69.19
Contraceptive prevalence rate (any method)	14.47	14.49
Immunization coverage, measles	52.20	38.95
% urban population	25.70	32.84
Total fertility rate	6.24	6.36
Total population (logged)	17.89	15.22
HIV prevalence, total (% of population ages 15–49)	4.58	3.23
Female labor force participation	49.47	51.13
GDP per capita (current US$)	398.76	476.08
Polity5 score	-1.16	-1.80
Mean years of schooling for females	2.32	1.98
WRA Mortality Rate 2004	7.32	7.43
WRA Mortality Rate 1997	7.63	7.68
WRA Mortality Rate 1990	6.86	6.71

Notes: USG: The United States government; ODA: overseas development assistance; USD: United States dollar; HIV: human immunodeficiency virus; GDP: gross domestic product; Polity5: policy score by INSCR; WRA: women of reproductive age

Figure S10. Trends in mortality rate among WRA from the optimized SCA for Scenario 2

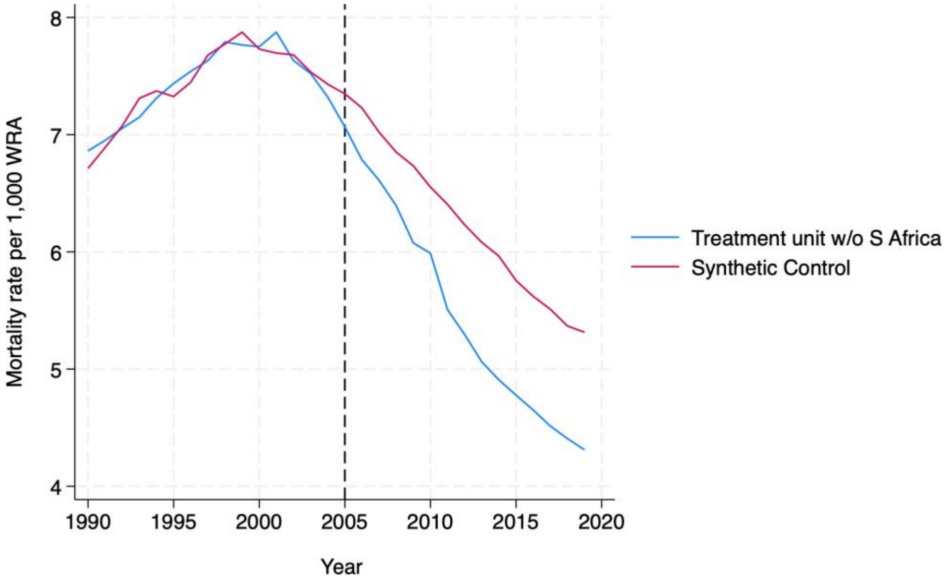

Figure S11. Effect estimates during the study period for Scenario

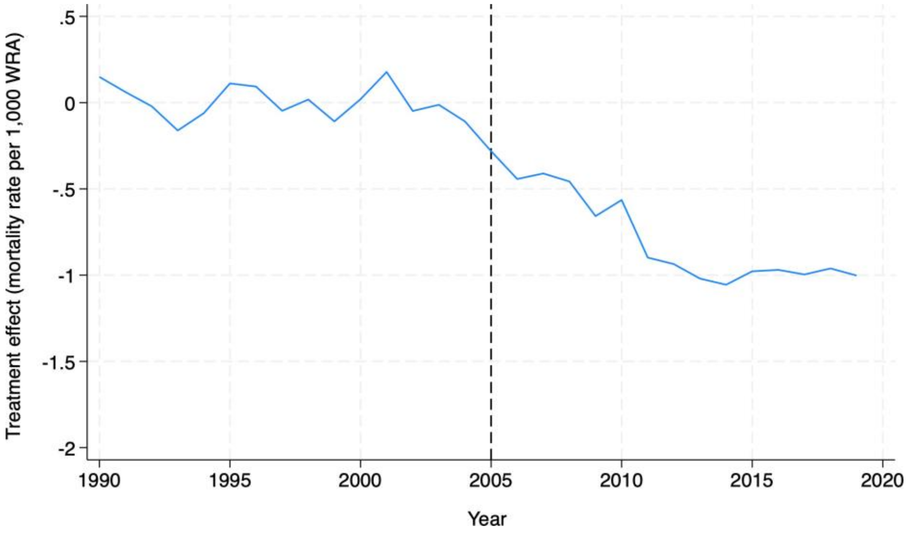

Table S4. Effect estimates and their p-values for Scenario 2

Year	Effect estimate	p-value	Standardized p-value*
2005	-0.2812	0.421	0.105
2006	-0.4426	0.316	0.053
2007	-0.4107	0.421	0.105
2008	-0.4570	0.368	0.105
2009	-0.6577	0.368	0.105
2010	-0.5645	0.368	0.105
2011	-0.8980	0.263	0.000
2012	-0.9360	0.211	0.000
2013	-1.0198	0.211	0.000
2014	-1.0558	0.211	0.000
2015	-0.9779	0.263	0.053
2016	-0.9695	0.316	0.053
2017	-0.9961	0.263	0.053
2018	-0.9612	0.316	0.053
2019	-1.0025	0.263	0.053
**Post-Treatment Average**	**-0.7754**	**0.316**	**0.053**

* p-value adjusted after accounting for pre-treatment matching quality

Our SCA model's fit for scenario 3 (i.e., without Nigeria) was also excellent (RMSPE = 0.0426896), as seen in Figure S12. The resulting synthetic control was a weighted average of the outcomes in the Central African Republic (48.3%), Gambia (20.9%), Chad (11.4%), Peru (7.6%), Sudan (2.3%), Eritrea (1.5%), Libya (1.2%), Niger (0.5%), Congo (0.1%), and Gabon (0.007%). Overall, the comparison of predictors used for SCA indicated a good resemblance of pre-intervention predictors between the treatment unit and

its synthetic control as well (see Table S5). During the post-intervention period, the mortality rate among WRA in the treatment group of countries decreased noticeably faster than the synthetic control (see Figure S12), and the average effect estimate (-1.12) was substantially larger than our original SCA. From the placebo test, we see that the effect estimates remain statistically significant (p<0.001) for the entire post-treatment period except 2005 (see Table S6).

Table S5. Predictor means between the treatment unit (without Nigeria) and synthetic control

**Predictors**	**Treatment unit w/o Nigeria**	**Synthetic control**
Net non-USG ODA received (current $US) per capita	26.39	32.75
Tuberculosis case detection rate	45.94	45.87
Contraceptive prevalence rate (any method)	19.97	18.14
Immunization coverage, measles	60.49	56.04
% urban population	24.96	39.30
Total fertility rate	5.91	5.90
Total population (logged)	17.23	15.16
HIV prevalence, total (% of population ages 15–49)	6.30	3.88
Female labor force participation	57.20	47.47
GDP per capita (current US$)	649.63	594.48
Polity5 score	-0.05	0.34
Mean years of schooling for females	2.69	1.84
WRA Mortality Rate 2004	7.84	7.84
WRA Mortality Rate 1997	7.08	7.09
WRA Mortality Rate 1990	5.90	5.87

Figure S12. Trends in mortality rate among WRA from the optimized SCA for Scenario 3

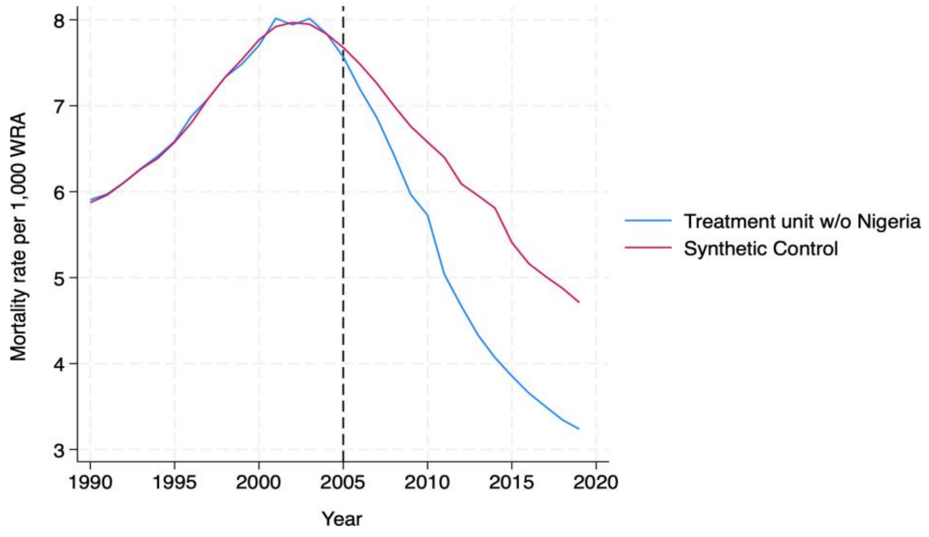

Figure S13. Effect estimates during the study period for Scenario 3

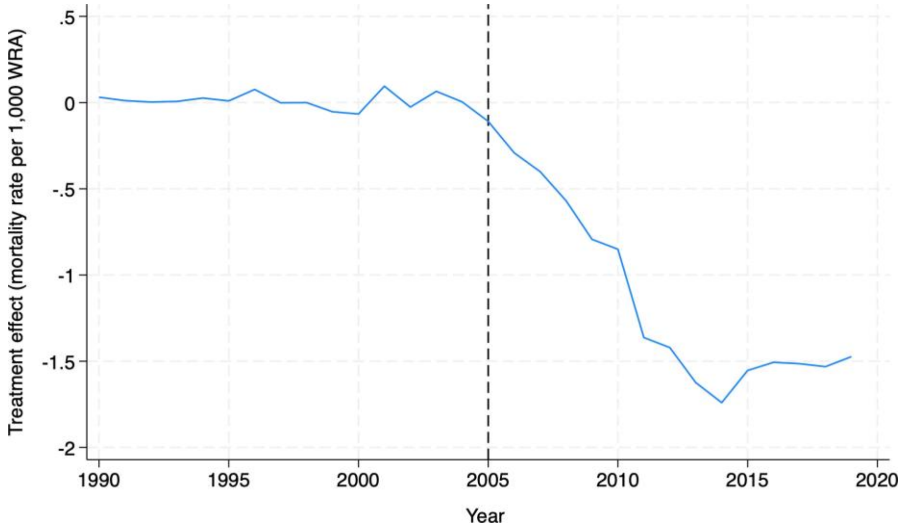

Table S6. Effect estimates and their p-values for Scenario 3

Year	Effect estimate	p-value	Standardized p-value*
2005	-0.1090	0.579	0.158
2006	-0.2912	0.474	<0.001
2007	-0.4003	0.421	<0.001
2008	-0.5690	0.368	<0.001
2009	-0.7931	0.211	<0.001
2010	-0.8505	0.368	<0.001
2011	-1.3627	0.105	<0.001
2012	-1.4208	0.105	<0.001
2013	-1.6238	0.105	<0.001
2014	-1.7403	0.105	<0.001
2015	-1.5535	0.105	<0.001
2016	-1.5063	0.105	<0.001
2017	-1.5141	0.105	<0.001
2018	-1.5314	0.105	<0.001
2019	-1.4734	0.053	<0.001
**Post-Treatment Average**	**-1.1159**	**0.158**	**<0.001**

* p-value adjusted after accounting for pre-treatment matching quality

Text S4. Residual analysis to examine the role of non-USG funding

In order to disentangle the effects of non-USG donor funding from USG funding, we conducted a residual analysis based on methods proposed by Arkhangelsky et al. (2021) and Clarke et al. (2023). The residual analysis is based on a two-step procedure.

Step 1: Estimating Residual Mortality
In the first step, we estimated a residual WRA mortality indicator that accounts for non-USG funding per capita. Let *i* denote county, *t* denote time in years, WRA mortality rate be denoted by (Y_{it}). The residual mortality variable (Y_{it}^{residual}) that controls for non-USG funding per capita ($X_{it.}$) is then estimated as follows:

$$Y_{it}^{\text{residual}} = Y_{it} - X_{it}\beta^{\&},$$

where $\beta^{\&}$ comes from an Ordinary Least Squares (OLS) regression of Y_{it} on X_{it}.

Step 2: Analyzing Residual ATE
In the second step, we use Y_{it}^{residual} as the outcome variable and re-run the same Synthetic Control Analysis (SCA) and Bayesian analyses (using the full list of predictors) as performed in the baseline models. This generates a "residual ATE" (Average Treatment Effect). We then compare the residual ATE to the base ATE obtained from the initial model, where the original WRA mortality/Y_{it} indicator was used as the outcome variable. To evaluate the influence of non-USG funding on WRA mortality, we compute the ratio as follows:

$$Ratio = \frac{Residual\ ATE}{Base\ ATE}$$

Interpreting the Ratio:

- **Ratio = 1**: Non-USG funding does not bias the main results. The observed effects are likely not influenced by non-USG funding.
- **Ratio < 1**: Non-USG funding may be contributing to the observed effects. A ratio of 0 suggests that the main results could be driven by non-USG funding or by USG funding's influence on non-USG funding, whereas a ratio of 0 to 1 suggests partial contribution of non-USG.
- **Ratio > 1**: Non-USG funding may be causing an underestimation of the effects of USG funding in the baseline analysis. This *suggests* that the potential effect of USG funding could be larger than initially estimated from base ATE.

By comparing the residual ATE to the base ATE, we assess whether non-USG funding affects the observed WRA mortality reductions. This analysis helps determine if the results from the main analysis are confounded by non-USG funding. Below are the findings of this sensitivity analysis, which suggests that non-USG funding is not greatly contributing to the observed mortality reductions in treatment countries, as seen by the ratios that are greater than one.

Table S7. Sensitivity Analysis of Average Treatment Effects for Residual and Base Models

Method	Treatment Effect	Including all Treatment Countries (n=16)			Excluding Nigeria (n=15)			Excluding South Africa (n=15)		
		ATE	*LB*	*UB*	*ATE*	*LB*	*UB*	*ATE*	*LB*	*UB*
Bayesian	Residual ATE	-1.74	-0.74	-2.73	-1.75	-0.73	-2.79	-0.86	0.02	-1.79
	Base ATE	-1.29	-0.18	-2.40	-1.73	-0.72	-2.71	-1.19	-0.20	-2.18
	Ratio	1.35	4.17	1.14	1.02	1.01	1.03	0.72	-0.11	0.82
Synthetic Control	Residual ATE	-0.69	X	X	-1.20	X	X	-0.73	X	X
	Base ATE	-0.66	X	X	-1.15	X	X	-0.85	X	X
	Ratio	1.04	X	X	1.04	X	X	0.87	X	X

Note: ATE: Average treatment effect; LB: Lower bound of 90% credible interval; UB: Upper bound of 90% credible interval.

Text S5. Findings from additional sensitivity analyses

Due to a gap in USAID's reported funding data for 2005 and 2006, we conducted additional sensitivity analyses using SCA and varied the treatment year. The average post-treatment effect estimate from SCA, considering 2007 as the treatment year instead of 2005, is statistically significant (p<0.001) but about 34% smaller than the effect estimate from our original SCA (see Table S7-1). Conversely, the average post-treatment effect estimate from SCA, considering 1999 as the treatment year, is about a quarter (24%) of the effect estimate from our original SCA (see Table S7-2). These findings reinforced our decision to select 2005 as the treatment year for evaluating the impact of USAID's health investment in LMICs.

Table S8. Effect estimates and their p-values for SCA considering 2007 as the treatment year

Year	Effect estimate	p-value	Standardized p-value*
2007	-0.0488	0.722	0.500
2008	-0.0838	0.722	0.278
2009	-0.2580	0.500	0.000
2010	-0.2124	0.611	0.167
2011	-0.6000	0.389	0.000
2012	-0.6137	0.278	0.000
2013	-0.7091	0.333	0.000
2014	-0.7556	0.333	0.000
2015	-0.5254	0.389	0.000
2016	-0.4694	0.444	0.000
2017	-0.4476	0.444	0.000
2018	-0.4396	0.389	0.000
2019	-0.4001	0.444	0.000
**Post-Treatment Average**	**-0.4279**	**0.444**	**<0.001**

* p-value adjusted after accounting for pre-treatment matching quality

Table S9. Effect estimates and their p-values for SCA considering 1999 as the treatment year

Year	Effect estimate	p-value	Standardized p-value*
1999	-0.1795	0.471	0.000
2000	-0.0957	0.882	0.000
2001	0.0661	0.824	0.353
2002	0.0277	1.000	0.882
2003	0.1288	0.882	0.059
2004	0.1633	0.765	0.000
2005	0.1257	0.824	0.059
2006	0.0613	1.000	0.588
2007	0.0806	0.941	0.588
2008	0.0577	1.000	0.765
2009	-0.1150	1.000	0.353
2010	-0.0701	1.000	0.706
2011	-0.4429	0.647	0.000
2012	-0.4616	0.588	0.000

2013	-0.5575	0.529	0.000
2014	-0.6021	0.529	0.000
2015	-0.3481	0.647	0.000
2016	-0.2939	0.706	0.059
2017	-0.2700	0.824	0.000
2018	-0.2606	0.882	0.000
2019	-0.2305	0.941	0.059
**Post-Treatment Average**	**-0.153**	**0.824**	**<0.001**

* p-value adjusted after accounting for pre-treatment matching quality
